# Sustainability of implementation of health-promotion practice in primary healthcare: a non-randomized parallel group study

**DOI:** 10.1186/s12913-026-15103-y

**Published:** 2026-07-20

**Authors:** Ylva Nilsagård, Emma Nilsing-Strid

**Affiliations:** 1 University Health Care Research Centre, Faculty of Medicine and Health, Örebro University, Örebro, SE-701 82 Sweden; 2 Department of Occupational and Environmental Medicine, Faculty of Medicine and Health, Örebro University, Örebro, SE- 701 82 Sweden

**Keywords:** Primary healthcare, Health promotion, Healthy lifestyle, Implementation science, Change management, Sustainability, Clinical practice guidelines

## Abstract

**Background:**

Despite the importance of promoting healthy lifestyles in primary healthcare (PHC) to prevent non-communicable diseases, implementing and sustaining evidence-based health-promotion lifestyle practices remains challenging. This study aimed to evaluate the sustainability of uptake of a health-promoting practice using a 12-month multifaceted implementation intervention in a Swedish PHC setting.

**Methods:**

A non-randomized parallel group design was used to compare five PHC intervention centers and five matched control centers with respect to health-promotion activities registered in medical records at: 6 months pre-implementation, during a 12-month implementation phase, and a follow-up at 18 months (sustainability). The intervention centers received a multifaceted implementation intervention based on a leadership change model using internal and external facilitators. Uptake was analysed using negative binomial mixed-effects models with a log link, modelling monthly uptake rates with an offset for the number of visits. Time since intervention initiation and time since the post-implementation phase were modelled using restricted cubic splines, allowing intervention effects to vary over time. Models were adjusted for seasonality, secular trends, patient sex, and site pair, with site included as a random effect. Intervention effects were estimated as ratios of rate ratios with simultaneous 95% confidence intervals.

**Results:**

The intervention centers successfully adopted and sustained the clinical intervention. At the 18-month follow-up, intervention centers sent out 7.2 times as many lifestyle screening forms compared with the control centers. The mean crude uptake difference was 43.6 and the relative rate was estimated at 2.23, indicating that patients at the intervention centers were more often asked about their lifestyle and more often received advice or consultative conversations about lifestyle changes.

**Conclusions:**

This multifaceted implementation intervention, focusing on leading change and facilitation in a routine clinical setting, increased the uptake of a health-promoting practice at the PHC intervention centers that was sustained over time. Health-promoting activities reached a larger proportion of patients in the intervention centers, indicating that the clinical intervention may work under routine conditions. These results are promising but need to be verified in larger randomized studies. In addition, differences between the intervention centers emphasize the need to explore the mechanisms of impact.

**Trial registration:**

This study was registered at ClinicalTrials.gov on 4 March 2021 (ref: NCT04799860).

**Supplementary Information:**

The online version contains supplementary material available at 10.1186/s12913-026-15103-y.

## Background

There is an ongoing paradigm shift in Swedish healthcare, moving from a healthcare system built up around diseases and institutions to a system designed for people. The aim is to develop a modern, equitable, accessible, and effective healthcare system with primary healthcare (PHC) as the hub [1]. This is intended to achieve a healthcare that focuses on the individual’s prerequisites and needs, builds upon relations, is proactive and health-promotive, contributes to equal health, security, and independence, and is founded in mutual responsibility and trust. One underlying reason for change is the challenge stemming from demographic development; fewer people available to provide care for people who live longer and with chronic (and often avoidable) diseases. A shift from reactive to proactive and health-promotive work is thus necessary to be able to meet the demands of the future. In line with this reform, PHC centers need to achieve a more health-promotive practice and use individually targeted interventions to address the increase in non-communicable diseases caused by unhealthy lifestyle habits [2].

Sweden has a national guideline that provides evidence-based recommendations with low risk of uncertainty regarding the advice that should be given to individuals in the case of tobacco use, harmful use of alcohol, low physical activity, and poor nutrition [3]. However, the uptake of such recommendations is commonly slow [4], and changes in practice are rarely accomplished simply by having access to published guidelines. In 2003, Grol and colleagues reported that around 30–40% of patients in the USA and the Netherlands did not receive care according to scientific evidence, and more than 20% received either unnecessary or even potentially harmful care [5]. Braithwaite and colleagues followed up the work of Grol, and reported in 2020 that only about 60% of healthcare was in line with evidence-based or consensus-based guidelines, whereas 30% was regarded as either wasted or of low value, and 10% was actually harmful [6]. In Sweden, only 1–7% of patients having unhealthy lifestyle habits and being at risk for non-communicable diseases are provided lifestyle counselling in PHC [7], illustrating the marked gap between guideline recommendations for health promotion and clinical practice.

There is a call for research on the uptake, implementation, and sustainability of health-promotion practices in healthcare, with a focus on behavior change in healthcare providers [8]. The Act in Time project [9], of which this study forms a part, strives to better understand how to support PHCs in achieving a more health-promoting practice in line with the recommendations in the national guideline [3]. The overall aim of the project is to design a generic implementation model to achieve a more proactive and health-promoting healthcare in PHC [9]. A pre-implementation phase of the project provided knowledge about the expectations of staff and managers in working towards a health-promotion practice, as well as their expectations regarding the support and readiness for change needed to achieve it [10, 11]. The effects of the implementation of the health promoting practice were assessed using a non‑randomized parallel‑group design, demonstrating that intervention centers yielded a higher relative uptake of health‑promotion activities than control centers, a difference that was sustained throughout a 6‑month post‑implementation period [12]. Once implemented, sustainability remains however a challenging matter [13, 14]. The initial enthusiasm may subside, and the change gradually peter out [15]. When the implementation support is withdrawn, it is up to the system to sustain the change and possibly further develop positive changes [16]. There is no complete agreement on the definition of sustainability, but Moore et al. developed a comprehensive definition including five constructs [17]: (i) after a defined period of time, (ii) the program, clinical intervention, and/or implementation strategies continue to be delivered and/or (iii) individual behavior change (i.e., clinician, patient) is maintained; (iv) the program and individual behavior change may evolve or adapt while (v) continuing to produce benefits for individuals/systems. The timeframe should preferably be at least a year post-implementation [15] and the need to withstand both internal and external challenges over time is also emphasized [18].

Working in a knowledge-intense organization such as healthcare is challenging when it comes to adhering to new knowledge that requires change of behavior in clinical practice. The difficulty of implementing new evidence in clinical practice is often underestimated [19–21], and structured support is seldom provided. General tools to support sustainability include written routines and quick reference guides, uncomplicated documentation processes, and easily accessible run-charts to visualize real-time results. The role of all leaders in the system is vital, not least in reviewing and discussing the performance using recent statistics and allowing time to address potential problems. Organizational leadership is also one of the most frequently reported facilitating factors for sustainment [22–24]. However, up to 70% of organizational change is not sustained [25]. This is particularly relevant for health-promotion interventions, as few are sustained beyond their initial implementation period, and active planning for sustainability is warranted [23].

Sustainability has been identified as one of the most critical gaps in implementation science [26]. Although there has been an increase in publications applying a sustainability approach during recent decades [24, 27, 28], scientific literature reporting the sustainability of practice change using independent observations is scarce [13]. The focus is often on sustainability determinants rather than evaluating sustainability outcomes, and quantitative research designs are limited [29, 30]. Furthermore, implementation interventions are seldom directed to healthcare providers and rarely use system-level outcomes [31], and there is a lack of studies evaluating sustainability of uptake of health-promoting practices in PHC.

The aim of the present study was to evaluate the sustainability of uptake of a clinical intervention using a multifaceted implementation intervention to achieve a health-promoting practice in a PHC setting. We hypothesized that the rate of health-promotion activities, as shown by administrative registration of advice, consultative conversations or qualified consultative conversations, physical activity prescriptions, and proportion of lifestyle screening forms sent out to patients would remain higher in the intervention centers compared to the control centers at the 18-month follow-up, manifesting as significantly higher absolute and relative effects.

## Methods

### Design

This study is part of the Act in Time project, which is registered at ClinicalTrials.gov (ref: NCT04799860). A non-randomized parallel group study design was used to compare intervention and matched control centers with respect to health-promotion activities registered in medical records at different time points: 6 months pre-implementation, during a 12-month implementation phase, and at follow-up phases at 6 months (effect) [12] and 18 months (sustainability). The sustainability of effects at 18-months follow-up is presented in this study. A sample size calculation was performed based on an expected minimum change of 10% from baseline to the 6-month follow-up, assuming a base rate (monthly health-promotion activities in controls) of 100 and an estimated 20% proportion of variability. For a one-tailed test, 9 PHCs were required to achieve 80% power (alpha 0.05).

### Setting

The study was conducted in Region Örebro County, in central Sweden, which has approximately 307 000 inhabitants and a total of 29 PHCs. Taxes and governmental contributions are the main funding sources for the Swedish healthcare system. The PHCs in the region are financed according to a per-capita reimbursement model, with some adjustments for the population profile in each service area. All PHCs are obliged to work with systematic health promotion and disease prevention.

Five intervention PHCs and five control PHCs matched on socioeconomic status and location (rural vs. urban) were enrolled between May and December 2021 in the Act in Time project [9]. The inclusion criteria were that the manager was interested in trying to achieve a change towards a more health-promotive practice and willing to identify two to three staff members who could set aside four hours/week to act as internal facilitators, supporting the implementation at the PHC. The study was authorized by the Ethical Review Authority, Sweden (refs: 2020–06956 and 2022-06918-02) and complied with the Helsinki Declaration. The Standards for Reporting Implementation Studies (StaRI) checklist [32] was followed.

### The clinical intervention

**Fig. 1:**
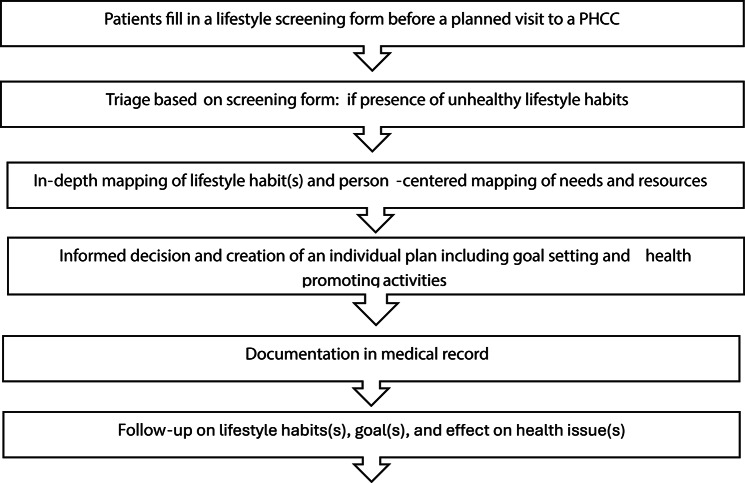
Description of the clinical health-promotion intervention. The health-promoting activities include screening for unhealthy lifestyle habits (tobacco use, harmful use of alcohol, low physical activity, and poor nutrition) followed by lifestyle advice, consulting or qualified consulting conversation according to the national guidelines [3, 12]. PHCC=primary healthcare center

The clinical intervention consists of a set of health-promoting activities according to the national guidelines [3, 12], and described in detail previously [9]. Briefly, the activities include screening for unhealthy lifestyle habits (tobacco use, harmful use of alcohol, low physical activity, and poor nutrition) using lifestyle screening form, providing lifestyle advice, consulting or qualified consulting conversation, and/or a physical activity prescription (Fig. 1). All activities and decisions should be made in conjunction with the patient, followed up, and registered in the medical record. All staff at the PHC center providing patient care were expected to perform the health-promoting activities.

### The implementation intervention

The implementation intervention was based on a leading change process as described by Astrakan [33], and used internal (IF) and external facilitators (EF) [34, 35]. The process included four phases: insight (current and desired state, identification of problems and opportunities), analysis (competencies, new desired behavior, who will be affected), planning (motivation, competence, communication, overarching plan for change), and implementing (including follow-up, acting on learning, establishing a new current state). The leading change process adhered to self-determination theory, which explains how intrinsic motivation to participate in a change is enhanced by focusing on the three psychological needs of mastery/competence, autonomy, and relatedness [36]. The EFs were experienced in quality improvement work, and were trained by the project leader (Y.N.) in leading change. They worked in pairs, and provided structure and support to the PHC managers and the IFs. A more thorough description is available in a previously published article [12] .

As the last step in the multi-faceted implementation intervention, an action plan was designed by the EFs in collaboration with the researchers (Appendix 1). This plan was inspired by a resource from the NHS Scotland Quality Improvement Hub [37], and used input gained from the pre-implementation research results where the participants stressed the importance of support from top management as crucial to maintain change [10, 11]. The action plan was handed over to the managers by the EFs after the end of the 12-month implementation intervention during a structured discussion, emphasizing the managers’ responsibility to continue the work towards a health-promotive practice and sustain the achieved change in practice. In addition, to further facilitate sustainability, the two expert facilitators and researchers (Y.N. and E.N.S.) developed recommendations directed at the top management level as a suggested way to support the PHC managers (Appendix 2). This was based on information retrieved during the initial interviews and focus group discussions [10, 11], the abovementioned NHS Scotland resource [37], and leading change literature [33].

## Data collection

De-identified aggregated data on health-promotion activities registered by staff in the medical records and lifestyle screening forms sent out to patients were reported monthly for each PHC by a controller at the Healthcare Administration. The health-promotion activities (primary outcome) were simple advice, consultative conversation, or qualified consultative conversation on physical activity, diet, tobacco use, and alcohol use, as well as physical activity prescriptions. Data were collected monthly during the baseline phase (6 months), active implementation phase (12 months), and follow-up phase (18 months). In total, data were collected for 48 months including the summer months (June, July, and August) for eight centers and for 46 months for two centers, due to a change in the medical journal system. The last data were collected in August 2024. Due to the PHC centers’ need to prioritize urgent clinical issues during vacation periods, summer months were excluded from the analyses. Influential events in the region or events affecting the region were recorded by two of the researchers (Y.N., E.N.S.).

### Statistical analysis

Uptake was analyzed using generalized linear mixed-effects models with a negative binomial error distribution and a log link. Parameters were estimated by maximum likelihood. The outcome was monthly uptake, defined as the number of health-promotion activities delivered per month. To model uptake rates, the logarithm of the number of visits in each month was included as an offset.

Because the duration of the implementation and post-implementation phases varied across sites, two time variables were defined. Time since initiation of the intervention (time v1) and time since the start of the post-implementation phase (time v2) were modelled using restricted cubic splines with three knots placed at the lower, median, and upper quartiles. Negative values were set to zero prior to modelling, such that all baseline observations before the intervention had time v1 = 0, while time v2 modified the trajectory of time v1 only during the post-implementation period. In the present study, follow-up was extended to a maximum of 18 months after the intervention phase.

Intervention status (no/yes) was included as a categorical explanatory variable and was allowed to interact with both time v1 and time v2. Calendar month was modelled using a restricted cubic spline with three knots to account for seasonal variation. A spline model is a statistical technique used to describe nonlinear relationships between a predictor and an outcome. Instead of forcing the data to follow a single straight line or a simple curve, splines allow the relationship to bend smoothly at specific points called knots. A linear term for study time, scaled relative to the earliest observed month, was included to adjust for long-term secular trends. Patient sex and site pair were included as categorical fixed effects, and site was included as a random intercept to account for clustering.

From the fitted model, marginal mean uptake rates per 1000 visits and corresponding 95% confidence intervals were estimated for a representative site with baseline, intervention, and post-intervention phases of 6, 12, and 18 months, respectively. These estimates correspond to a typical site randomly drawn from the population and were averaged over sex and site pair, with calendar month and long-term time trend held at their mean values.

Intervention effects were quantified using model-based contrasts with 95% confidence intervals adjusted for simultaneous inference using the multivariate t distribution. On the log scale, the intervention effect corresponds to a difference-in-differences comparing changes from baseline between intervention and control groups. After exponentiation, this yields a ratio of rate ratios, which was used to summarize the intervention effect. Owing to the longitudinal design, intervention effects were presented as smooth functions of time with simultaneous 95% confidence intervals.

All analyses were conducted in R using the packages glmmTMB, DHARMa, emmeans, and the tidyverse. Full details of the modelling framework have been published previously, and the present analysis extends this approach through longer follow-up [12].

## Results

A total of 619 430 healthcare visits were registered in the medical records during the study period excluding June, July, and August: 293 050 at the intervention centers and 326 380 at the control centers (Appendix 3). All were used in the analyses. The intervention centers sent out a total of 5778 lifestyle screening forms, compared to 963 in the control centers (total 6741). At baseline, the control centers sent out six times as many forms per 1000 patient visits compared to the intervention centers. This shifted during the intervention period and the 18-month follow-up, where the intervention centers sent out 8.2 and 7.2 times as many forms per 1000 patient visits, respectively. In total, 22 105 health-promotion activities were registered in the medical records for the intervention centers, compared to 15 504 in the control centers during the study period (total: 37 609).

The control centers had slightly more healthcare visits than the intervention centers, and so mean uptake per 1000 visits and crude mean uptake differences are presented in Table 1. The results show that the intervention centers adopted the clinical intervention by increasing the mean uptake per 1000 visits during both the active implementation phase and the 18-month follow-up phase, providing health-promotive activities to a larger proportion of patients. Conversely, the control centers increased their mean uptake mainly during the follow-up phase. The crude mean uptake differences were in favor of the intervention centers at both active and follow-up phases.

**Table 1:** Healthcare visits, number of lifestyle screening forms, and health-promotion activities for the intervention and control centers at baseline, during the active phase, and during follow-up. Uptake refers to health-promotion activities registered in medical records per 1000 visits. The health-promotion activities include simple advice, consultative conversation, or qualified consultative conversation on physical activity, diet, tobacco use, and alcohol use, and physical activity prescriptions

Phase	Healthcare visits		Lifestyle screening forms		Health-promotion activities		Mean uptake per 1000 visits		Crude mean uptake difference
	Intervent.	Control	Intervent.	Control	Intervent.	Control	Intervent.	Control	
Baseline	42 436	48 204	32	202	1685	1800	39.7	37.3	2.4
Active	135 032	147 389	2 423	297	8720	6171	64.6	41.9	22.7
18-month follow-up	115 582	130 787	3323	464	11 700	7533	101.2	57.6	43.6

**Fig. 2:**
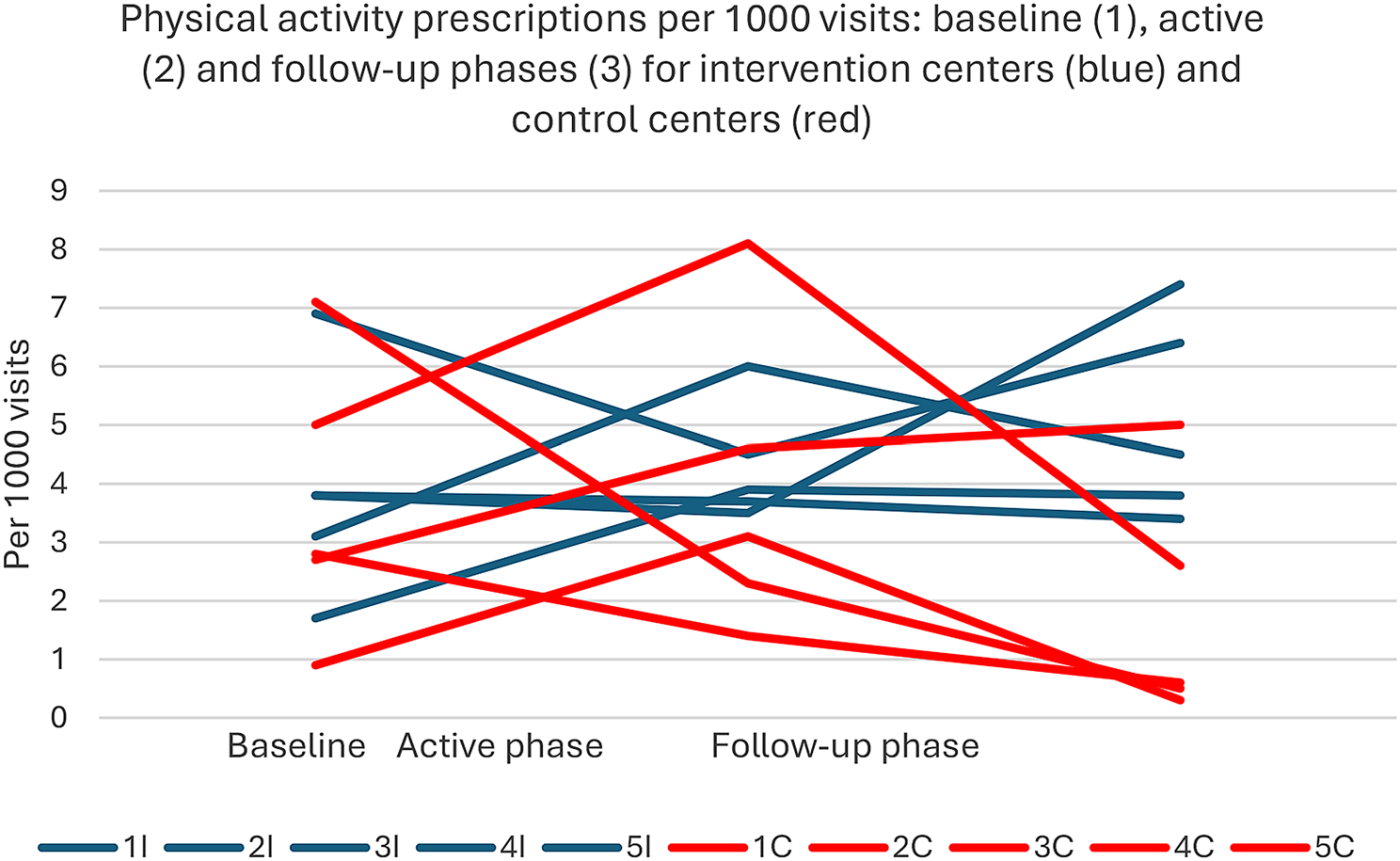
Physical activity prescriptions per 1000 patient visits: (1) at baseline, (2) during the active phase, and (3) during follow-up. Intervention centers are shown in blue (1I–5I) and control centers are shown in red (1–5 C)

The intervention centers prescribed physical activity to a higher extent (*n* = 1473) than the control centers (*n* = 970): 191 versus 146 at baseline, 608 versus 556 during the active phase, and 674 versus 268 during the follow-up phase. In terms of prescriptions per 1000 patient visits, all but one of the control centers prescribed physical activity to a lesser extent during follow-up than they had at baseline. Three intervention centers increased their prescription, while one prescribed less and another one prescribed marginally less (Fig. 2 and Appendix 4).

**Fig. 3:**
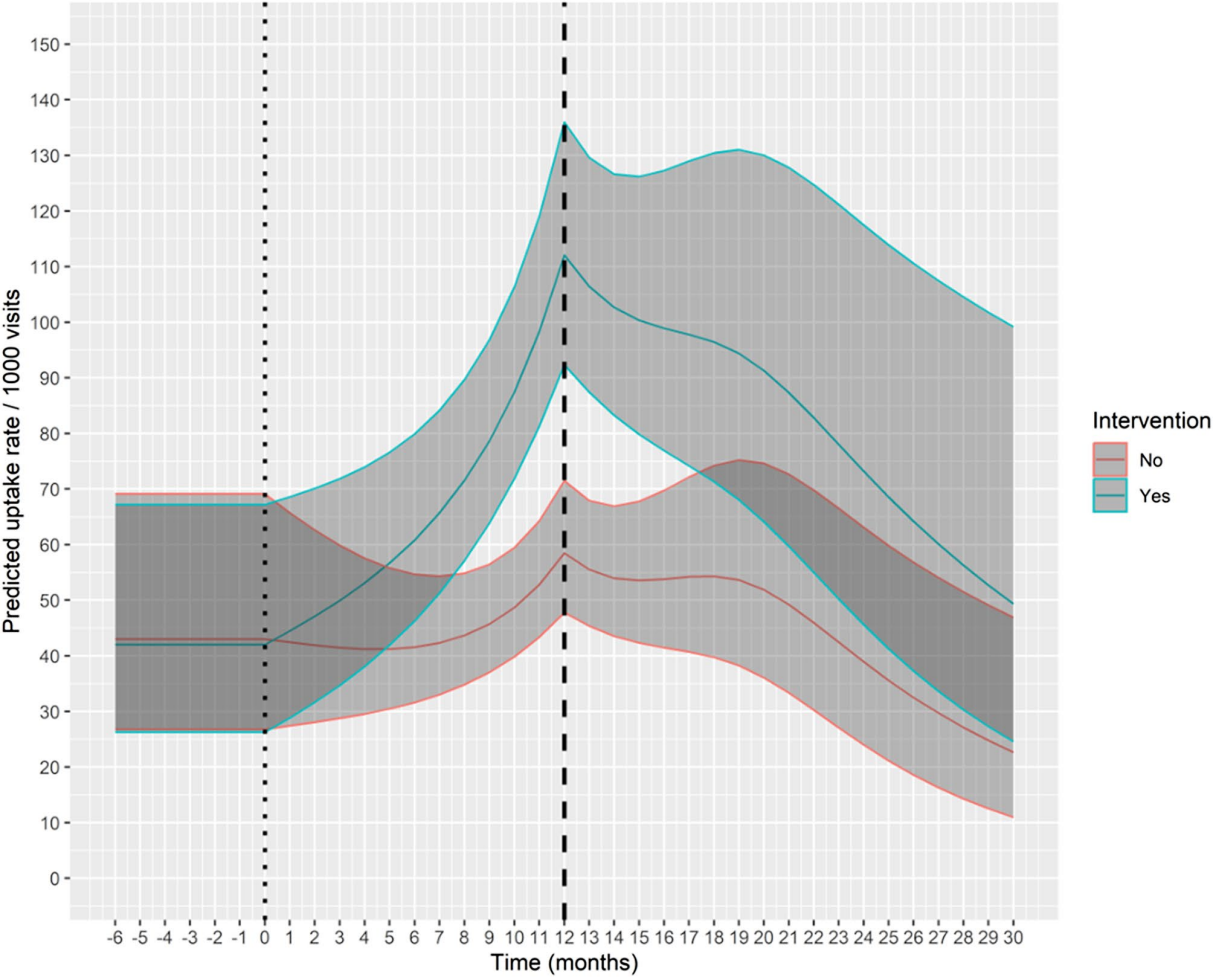
Predicted uptake rate of health-promoting activities per 1000 visits for baseline, active implementation intervention and follow-up phases for the intervention and control centers. Dotted line: baseline; dashed line: end of active implementation phase; shaded areas: 95% confidence intervals

To estimate the effect of the implementation intervention, uptake was modeled using generalized linear mixed models. Figure 3 shows the model-based predicted mean uptake rate for health-promotion activities for the intervention and control centers with baseline, active, and follow-up phase lengths of 6, 12, and 18 months, respectively. The uptake rate increased exponentially from the first month into the active phase until ending of implementation support. A statistically significant effect on uptake was present from month 7 of the implementation support to the first 6 months of follow-up. Although the trend line shows a general decline in the post-implementation phase for the treatment group, the wide confidence intervals do not rule out a plateauing effect. Confidence intervals are given in Appendix 5.

**Fig. 4:**
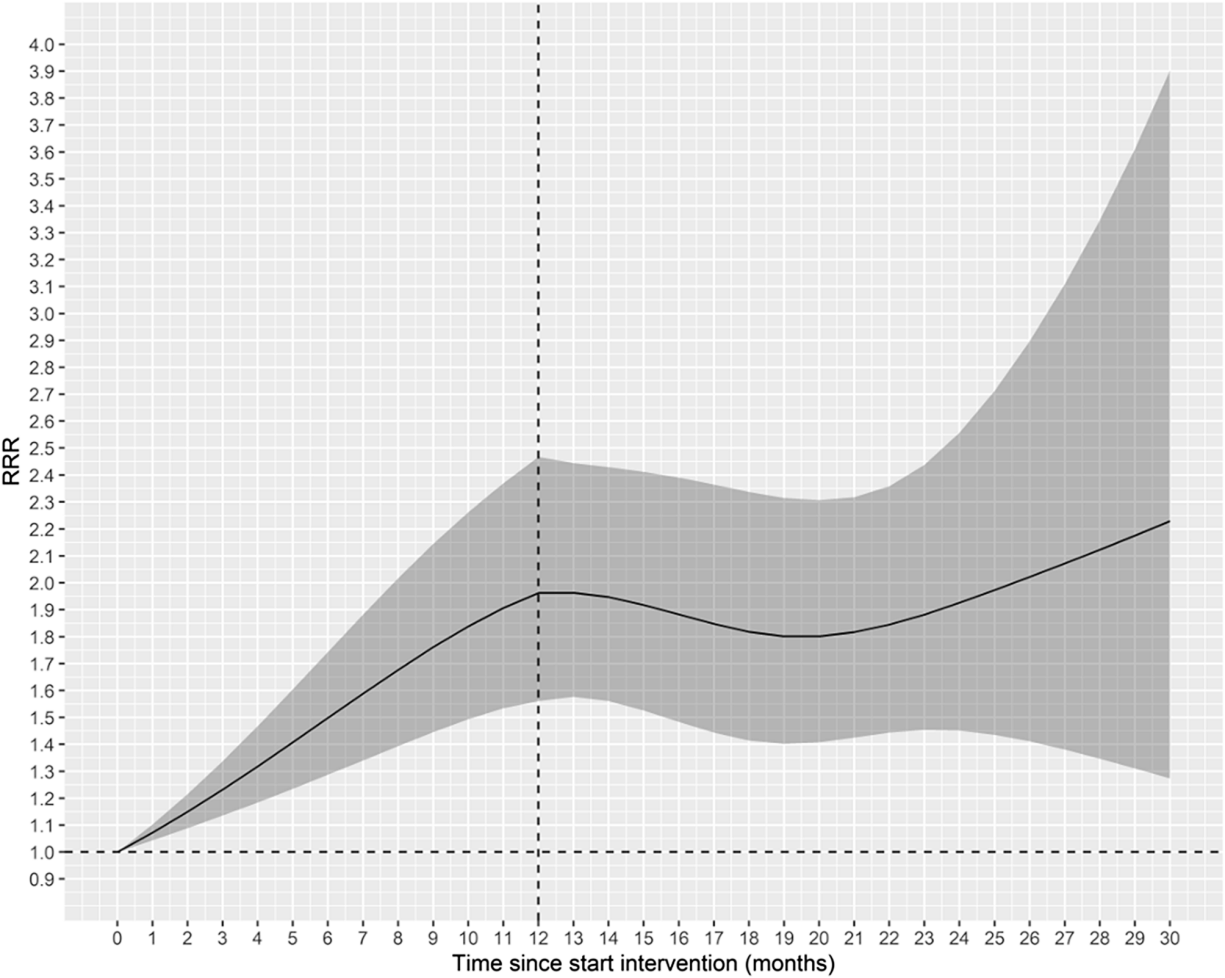
Relative implementation intervention effect over the 12-month active implementation phase (adoption of the health-promotive intervention) and the 18-month follow-up phase (sustainability). Solid line: RRR, Rate of rate ratios; shaded area: 95% confidence intervals. Values > 1 represent higher uptake in the intervention group

Figure 4 presents the relative implementation intervention effect, expressed as the ratio of rate ratios (RRR), during the active implementation phase (adoption of the clinical health-promotive intervention) and the subsequent 18-month follow-up phase (sustainability). During the active implementation phase, the RRR increased approximately linearly. At 6 months, the RRR was 1.50 [1.29, 1.74], indicating that uptake in intervention centers was approximately 50% higher than in control centers. By 12 months, the RRR had increased to 1.96 [1.56, 2.47], corresponding to nearly a doubling of uptake relative to control centers. The confidence intervals are shown in Appendix 6. As the confidence intervals at both time-points exclude unity, the intervention effect during the active phase was statistically significant.

During the follow-up phase, the intervention effect was largely maintained. At the 18-month follow-up (month 30), the RRR was 2.23 [1.28, 3.90], indicating that uptake in intervention centers remained more than twice that observed in control centers. Although the fitted trend line suggests a very slight dip followed by further increases between approximately 20 and 30 months, confidence intervals widen during this period, and therefore a plateau in the sustained intervention effect cannot be ruled out.

## Discussion

The multifaceted implementation intervention evaluated in this study increased the uptake of a health-promoting practice at the PHC intervention centers, and this increase was sustained over time. At the 18-month follow-up, the mean crude uptake rate difference was 43.6 and the relative rate was estimated at 2.23. Patients were over 8 times per 1000 visits more likely to be asked to reflect on their lifestyle habits at the intervention centers compared to control centers during the active implementation phase, and this difference was well-sustained over the 18-month follow-up phase (7.2). With this implementation intervention, patients were more likely to be asked about their lifestyle and more often received advice, consultative conversations, or qualified conversations in line with the recommendations of the national guideline [3]. Patients visiting the intervention centers were also more likely to receive a prescription for physical activity. The increased focus on working with health-promotion may be a reason for the better results seen at the intervention centers. These findings contribute to the recognized research gap, including determining how health-promoting practices can be adopted and sustained in PHC [8]. The Act in Time project offers a comprehensive approach targeting the organizational level, where the implementation intervention supports the transition of knowledge into clinical practice and real utilization of the evidence; the evidence becomes effective and meaningful in the meeting with patients.

The need to work with health promotion is urgent, since half of all women and two-thirds of all men have at least one unhealthy lifestyle habit, children and adolescents are less physically active [38]. Health-promotion interventions encouraging healthy lifestyle habits in settings such as PHC are relevant and effective in improving individuals’ quality of life [39] and reducing disease-related risks, including cardiovascular diseases and mortality [40]. To reduce and control non-communicable diseases and improve health, integration of health promotion in PHC has been emphasized and clinical guidelines have been developed [3]. However, implementation of clinical guideline recommendations or evidence-based interventions implies a change in clinical practice, where the context in which the change takes place plays an important role [41–43].

Implementation strategies, defined as techniques used to enhance the adoption, implementation, and sustainability of a clinical intervention, constitute the “how-to” component of changing healthcare practice [44]. Once a guideline or evidence-based intervention is adopted and implemented in practice, the next challenge is to sustain the improvements over time, as people tend to fall back into old routines [15, 45]. In line with the findings from the Act in Time project [12], multifaceted implementation interventions may increase PHC professionals’ delivery of preventive care targeting unhealthy lifestyles, but the change of practice has rarely been followed up beyond 12 months [46].

In the few previous studies that have explicitly evaluated the sustainability of implementing health-promotion practices in PHC targeting different unhealthy lifestyle habits, the effects have been negligible [47, 48]. The long-term results of the Act in Time project therefore contribute with knowledge of how to sustain evidence-based health promotion in routine PHC practice by supporting the PHC centers, the managers, and the healthcare professionals in a theory-based multifaceted implementation intervention including an action plan for sustainability [49]. In the Act in Time project, the evaluation of sustainability was planned early on. Moreover, the action plan for sustainability was based on a quality improvement resource from NHS Scotland [37] and further informed by the results of the studies in the pre-implementation phase [10, 11] and discussions with the facilitators and managers. The goal was to tailor the plan according to contextual needs and thereby improve the possibility of implementation success [50, 51].

We argue that this study is an implementation effectiveness study, in line with the distinction between efficacy and effectiveness outlined by Nilsen et al. [52]. The implementation intervention was conducted in a routine clinical setting with existing staff and infrastructure, and minimal extra support provided by EFs and IFs. The IFs set time aside to support the implementation during the active implementation phase, but the resources were supplied by the PHC centers to enable ownership and sustainable change in practice. In addition, the population of interest was diverse, consisting of PHC centers with variable levels of readiness, leadership, and capacity. This will be further explored in a process evaluation study.

The goal was to evaluate whether the intervention succeeds under real-world conditions, not ideal conditions, and so the intervention was directed to all PHC professions to evaluate whether the clinical intervention worked in routine praxis [52]. The implementation intervention showed promising sustainability over 18 months despite real-world challenging and potentially influencing events such as organizational, financial, and administrative matters. At the time this study started, restrictions due to the COVID-19 pandemic were still in place but the peak of infections had passed. During the study period, there were organizational changes within the Integrated Care Division responsible for the regional PHC. Due to the strained financial situation of the Health and Medical Administration in Region Örebro and preparations for a new medical record system, only operationally critical staff training was allowed from March 2023 to 2025. This led to withdrawal of all staff training on how to advise or counsel patients about tobacco use, harmful use of alcohol, low physical activity, or poor diet. At the same time, healthcare professionals and managers were encouraged to consider whether the care they provided was necessary, urgent, or desirable. There were intense discussions on how to look upon health promotion in healthcare: for whom, and for how long? Between January and March 2024, all staff in the region were trained to use the new medical record system that was introduced in September 2024. All the factors mentioned above can be interpreted as interacting negatively with the possibility of adopting a health-promotive PHC practice. During the study period, there were also changes in managers and staff turnover was constantly ongoing, which may hamper the study of implementation effect and sustainability in healthcare.

Continuation of providing evidence-based interventions can be measured as sustainability outcomes of implementation strategies [53], but evaluations of sustainability of practice change including objective measurements are rare, and seldom directed towards healthcare professionals or system-level outcomes [13]. In the present study, we investigated the extent to which health-promoting activities continued to be delivered over an 18-month follow-up using aggregated and de-identified medical record data collected from intervention and control centers. The findings indicate that the health-promoting practice might have become institutionalized [17, 53] at the intervention centers.

There were, however, differences in success between the intervention centers, despite tailored implementation support, and the underlying causes will later be evaluated by combining qualitative and quantitative data. Experiences of the implementation of the health-promoting practice and sustainability of behavior change from a healthcare professional level will be described in a qualitative study (submitted). Top managers’ and managers’ experiences of being responsible for the sustainability of the health-promotion practice over time and their compliance with the action plan and checklist will also be further explored within the Act in Time project. This multifaceted implementation intervention showed promising effect over time, but for increased knowledge of how to integrate and sustain health-promotion practice within routine PHC, replications in other PHC settings are warranted [8]. Further research is also needed on how to scale up a healthy-lifestyle-promoting practice in PHC.

### Methodological considerations

The strengths of this study include the large amount of data collected over 4 years, with the analysis covering a total of 36 months (summer months excluded). A further strength is the real-world context and the use of real-world data in the form of medical records [54] to evaluate change of practice. Medical record registration served as a proxy for behaviour change, although documentation and reporting bias cannot be excluded, as professionals in both intervention and control centers may provide more - or less - counselling than is recorded. Instead of a randomized controlled design, this study employed a longitudinal design including parallel comparisons using matched controls. The design is useful in examining the impact of a complex implementation intervention in a real-world setting when a randomized controlled trial is not feasible, and when assessing the adoption of and adherence to guideline recommendations by the healthcare system [55, 56]. Covariate adjustment and advanced modelling techniques such as regression splines were used for capturing non-linear associations. We also included testing of model assumptions and fit (Appendix 7).

Although the control centers were matched to the intervention centers, the absence of randomization indicates that selection bias cannot be ruled out and some caution is needed. When leading change, it is of utmost importance that the managers are willing to take on the responsibility as change owners [33]. Therefore, this was conditionally an inclusion criterion, together with the willingness to accept implementation support and identify 2–3 staff members who could set aside 4 h per week to act as IFs. In this real-world setting, all centers that were interested in being intervention centers were included as intervention centers, and other centers were used as matched controls.

The control centers increased their health-promoting practice on a curve like that seen for the intervention centers, although to a significantly lower extent. This might be a research participation effect [57], as throughout the study period, health promotion and the research project were discussed at managerial meetings at different levels in the region, and attention was given to the project via the local TV station. This means that some control centers may have tried to improve their practice by themselves.

Excluding summer months were excluded from the analysis due to the PHC centers’ need to focus on acute matters during the vacation period. This may have introduced bias and the results should be interpreted with some caution. During these months, the PHC centers had limited time to work actively on the implementation activities, and health-promotion activities were not prioritized when meeting patients. Despite this, a small increase in registered health-promotion activities was also noted in these months (Appendix 3).

Aggregated de-identified data were available from study start until the new medical record system was introduced. This resulted in two missed months of follow-up for one pair of healthcare centers (one intervention center and the matched control). The study period stretched over four years, and it was not possible to account for such contextual influencing factors.

## Conclusion

The present study shows that qualified long-term support facilitates the uptake of health-promotive practice in primary healthcare. Patients visiting the intervention centers were more likely to be sent lifestyle forms to answer before their healthcare visit, and more likely to receive lifestyle advice or counseling compared to patients visiting the control centers. This difference was still present 18 months after the end of implementation support. These findings imply that strategies such as using internal and external facilitators, relying on leading change models, using real-world data as outcomes, and planning for sustainability early on are beneficial. However, the mechanism of impact needs to be further studied, and larger randomized controlled trials are needed to verify the results.

## Supplementary Information

Below is the link to the electronic supplementary material.

Supplementary Material 1

Supplementary Material 2

Supplementary Material 3

Supplementary Material 4

Supplementary Material 5

Supplementary Material 6

Supplementary Material 7

## Data Availability

The datasets supporting the conclusions of this article are included within the article and its additional files.

## Abbreviations

EF: External facilitator
IF: Internal facilitator
PHC: Primary healthcare
PHCC: Primary healthcare center

## References

[1] The ministry of health and social affairs (Socialdepartementet). Summary of good quality, local health care. The right support for mental health. SPU. 2021:6. Stockholm: the ministry of health and social affairs. Available from: https://www.government.se/legal-documents/2021/02/summary-of-good-quality-local-health-care.-the-right-support-for-mental-health-sou-20216. Accessed 27 May 2026. 2021.

[2] World Health Organization. Noncommunicable diseases 2025 [updated September 25]. Available from: https://www.who.int/news-room/fact-sheets/detail/noncommunicable-diseases

[3] National Board of Health and Welfare (Socialstyrelsen). Nationella riktlinjer för prevention och behandling vid ohälsosamma levnadsvanor - stöd för styrning och ledning. Socialstyrelsen. 2018. (In Swedish). Available from https://www.socialstyrelsen.se/kunskapsstod-och-regler/regler-och-riktlinjer/nationella-riktlinjer/riktlinjer-och-utvarderingar/levnadsvanor/. Accessed 27 May 2026.

[4] Kardakis T, Jerdén L, Nyström ME, Weinehall L, Johansson H. Implementation of clinical practice guidelines on lifestyle interventions in Swedish primary healthcare - a two-year follow up. BMC Health Serv Res. 2018;18(1):227. 29606110 10.1186/s12913-018-3023-z PMC5880081

[5] Grol R, Grimshaw J. From best evidence to best practice: effective implementation of change in patients’ care. Lancet. 2003;362(9391):6. 10.1016/S0140-6736(03)14546-1 14568747

[6] Braithwaite J, Glasziou P, Westbrook J. The three numbers you need to know about healthcare: the 60-30-10 Challenge. BMC Med. 2020;18(1):102. 32362273 10.1186/s12916-020-01563-4 PMC7197142

[7] National Board of Health and Welfare. (Socialstyrelsen). Primärvårdens stöd till patienter med ohälsosamma levnadsvanor. Socialstyrelsen. 2021. (In Swedish). Available from: https://www.socialstyrelsen.se/publikationer/primarvardens-stod-till-patienter-med-ohalsosamma-levnadsvanor-2023-2024-11-9306/. Accessed 27 May 2026.

[8] Vos RC, van Osch L, van Bilsen JHM, Knapen MJ, Evers AWM, Hopman MTE, et al. Evidence-based implementation of lifestyle medicine in healthcare practice: a research agenda. Fam Med Community Health. 2025;13(3). 10.1136/fmch-2025-003324 PMC12406807 40889887

[9] Strid-Nilsing E, Wallin L, Nilsagård Y. Implementation of a health promotion practice using individually targeted lifestyle interventions in primary health care: protocol for the Act in time mixed methods process evaluation study. JMIR Res Protoc. 2022;11(8):e37634. 35984700 10.2196/37634 PMC9440414

[10] Strid-Nilsing E, Wallin L, Nilsagård Y. Expectations on implementation of a health promotion practice using individually targeted lifestyle interventions in primary health care: a qualitative study. BMC Prim Care. 2023;24(1):122. 37328813 10.1186/s12875-023-02079-5 PMC10273504

[11] Strid-Nilsing E, Wallin L, Nilsagård Y. Exploring expectations and readiness for healthy lifestyle promotion in Swedish primary health care: a qualitative analysis of managers, facilitators, and professionals. Scand J Prim Health Care. 2024;42(1):13. 10.1080/02813432.2023.2301556 PMC10851800 38241166

[12] Nilsagård YE, Smith DR, Söderqvist F, Strid-Nilsing E, Wallin L. Achieving health-promotion practice in primary care using a multifaceted implementation strategy: a non-randomized parallel group study. Implement Sci Commun. 2025;6(1):36. 40197376 10.1186/s43058-025-00723-y PMC11977894

[13] Shelton RC, Cooper BR, Stirman SW. The Sustainability of evidence-based interventions and practices in public health and health Care. Annu Rev Public Health. 2018;39:55–76. 29328872 10.1146/annurev-publhealth-040617-014731

[14] Silver SA, McQuillan R, Harel Z, Weizman AV, Thomas A, Nesrallah G, et al. How to sustain change and support continuous quality improvement. Clin J Am Soc Nephrol. 2016;11(5):916–24. 27016498 10.2215/CJN.11501015 PMC4858491

[15] Wiltsey Stirman S, Kimberly J, Cook N, Calloway A, Castro F, Charns M. The sustainability of new programs and innovations: a review of the empirical literature and recommendations for future research. Implement Sci. 2012;7:17. 22417162 10.1186/1748-5908-7-17 PMC3317864

[16] Chambers DA, Glasgow RE, Stange KC. The dynamic sustainability framework: addressing the paradox of sustainment amid ongoing change. Implement Sci. 2013;8:117. 24088228 10.1186/1748-5908-8-117 PMC3852739

[17] Moore JE, Mascarenhas A, Bain J, Straus SE. Developing a comprehensive definition of sustainability. Implement Sci. 2017;12(1):110. 28865479 10.1186/s13012-017-0637-1 PMC5581411

[18] Maher L, Gustafson D, Evans A. Sustainability model and guide. NHS Institute for innovation and improvement; 2010.

[19] Nilsen P, Schildmeijer K, Ericsson C, Seing I, Birken S. Implementation of change in health care in Sweden: a qualitative study of professionals’ change responses. Implement Sci. 2019;14(1):51. 31088483 10.1186/s13012-019-0902-6 PMC6518624

[20] Wändell PE, de Waard AM, Holzmann MJ, Gornitzki C, Lionis C, de Wit N, et al. Barriers and facilitators among health professionals in primary care to prevention of cardiometabolic diseases: A systematic review. Fam Pract. 2018;35(4):383–98. 29385438 10.1093/fampra/cmx137

[21] Squires JE, Graham I, Bashir K, Nadalin-Penno L, Lavis J, Francis J, et al. Understanding context: A concept analysis. J Adv Nurs. 2019;75(12):3448–70. 31359451 10.1111/jan.14165

[22] Hailemariam M, Bustos T, Montgomery B, Barajas R, Evans LB, Drahota A. Evidence-based intervention sustainability strategies: a systematic review. Implement Sci. 2019;14(1):57. 31171004 10.1186/s13012-019-0910-6 PMC6554955

[23] Bodkin A, Hakimi S. Sustainable by design: a systematic review of factors for health promotion program sustainability. BMC Public Health. 2020;20(1):964. 32560718 10.1186/s12889-020-09091-9 PMC7304137

[24] Zurynski Y, Ludlow K, Testa L, Augustsson H, Herkes-Deane J, Hutchinson K, et al. Built to last? Barriers and facilitators of healthcare program sustainability: a systematic integrative review. Implement Sci. 2023;18(1):62. 37957669 10.1186/s13012-023-01315-x PMC10641997

[25] Beer M, Nohria N. Cracking the code of change. Harv Bus Rev. 2000;78(3):133–41. 11183975

[26] Johnson AM, Moore JE, Chambers DA, Rup J, Dinyarian C, Straus SE. How do researchers conceptualize and plan for the sustainability of their NIH R01 implementation projects? Implement Sci. 2019;14(1):50. 31072409 10.1186/s13012-019-0895-1 PMC6506963

[27] Lennox L, Linwood-Amor A, Maher L, Reed J. Making change last? Exploring the value of sustainability approaches in healthcare: a scoping review. Health Res Policy Syst. 2020;18(1):120. 33050921 10.1186/s12961-020-00601-0 PMC7556957

[28] Liu XL, Wang T, Tan JY, Stewart S, Chan RJ, Eliseeva S, et al. Sustainability of healthcare professionals’ adherence to clinical practice guidelines in primary care. BMC Prim Care. 2022;23(1):36. 35232391 10.1186/s12875-022-01641-x PMC8889781

[29] Flynn R, Stevens B, Bains A, Kennedy M, Scott SD. Identifying existing approaches used to evaluate the sustainability of evidence-based interventions in healthcare: an integrative review. Syst Rev. 2022;11(1):221. 36243760 10.1186/s13643-022-02093-1 PMC9569065

[30] Lennox L, Maher L, Reed J. Navigating the sustainability landscape: a systematic review of sustainability approaches in healthcare. Implement Sci. 2018;13(1):27. 29426341 10.1186/s13012-017-0707-4 PMC5810192

[31] Tricco AC, Ashoor HM, Cardoso R, MacDonald H, Cogo E, Kastner M, et al. Sustainability of knowledge translation interventions in healthcare decision-making: a scoping review. Implement Sci. 2016;11:55. 27097827 10.1186/s13012-016-0421-7 PMC4839064

[32] Pinnock H, Barwick M, Carpenter CR, Eldridge S, Grandes G, Griffiths CJ, et al. Standards for Reporting Implementation Studies (StaRI) Statement. BMJ. 2017;356:i6795. 28264797 10.1136/bmj.i6795 PMC5421438

[33] Odell K. Förändringshandboken för ledare och medarbetare. Stockholm: Liber AB; 2019.

[34] Eldh AC, Hälleberg Nyman M, Joelsson-Alm E, Wallin L. Facilitating facilitators to facilitate - some general comments on a strategy for knowledge implementation in health services. Front Health Serv. 2023;17(3). 10.3389/frhs.2023.1112936 PMC10149731 37138952

[35] Lessard S, Bareil C, Lalonde L, Duhamel F, Hudon E, Goudreau J, et al. External facilitators and interprofessional facilitation teams: a qualitative study of their roles in supporting practice change. Implement Sci. 2016;11:97. 27424171 10.1186/s13012-016-0458-7 PMC4947272

[36] Ryan RM, Deci EL. Self-determination theory and the facilitation of intrinsic motivation, social development, and well-being. Am Psychol. 2000;55(1):68–78. 11392867 10.1037//0003-066x.55.1.68

[37] Jeffcott S. The spread and sustainability of quality improvement in healthcare. A resource to increase understanding of the 10 key factors underpinning successful spread and sustainability of quality improvement in NHS Scotland. NHS Scotland Quality Improvement Hub; 2014.

[38] World Health Organization. Global action plan for the prevention and control of noncommunicable diseases 2013–2020. World Health Organization. 2013. Available from: https://www.who.int/publications/i/item/9789241506236. Accessed 27 May 2026.

[39] Amiri S, Mahmood N, Junaidi S, Khan MA. Lifestyle interventions improving health-related quality of life: A systematic review and meta-analysis of randomized control trials. J Educ Health Promot. 2024;13:193. 39268447 10.4103/jehp.jehp_1156_23 PMC11392327

[40] Bisak A, Stafström M. Unleashing the potential of health promotion in primary care-a scoping literature review. Health Promot Int. 2024;39(3). 10.1093/heapro/daae044 PMC11127486 38795052

[41] Damschroder LJ, Aron DC, Keith RE, Kirsh SR, Alexander JA, Lowery JC. Fostering implementation of health services research findings into practice: a consolidated framework for advancing implementation science. Implement Sci. 2009;4:50. 19664226 10.1186/1748-5908-4-50 PMC2736161

[42] May CR, Johnson M, Finch T. Implementation, context and complexity. Implement Sci. 2016;11(1):141. 27756414 10.1186/s13012-016-0506-3 PMC5069794

[43] Nilsen P, Bernhardsson S. Context matters in implementation science: a scoping review of determinant frameworks that describe contextual determinants for implementation outcomes. BMC Health Serv Res. 2019;19(1):189. 30909897 10.1186/s12913-019-4015-3 PMC6432749

[44] Proctor EK, Powell BJ, McMillen JC. Implementation strategies: recommendations for specifying and reporting. Implement Sci. 2013;8:139. 24289295 10.1186/1748-5908-8-139 PMC3882890

[45] Ament SM, de Groot JJ, Maessen JM, Dirksen CD, van der Weijden T, Kleijnen J. Sustainability of professionals’ adherence to clinical practice guidelines in medical care: a systematic review. BMJ Open. 2015;5(12):e008073. 26715477 10.1136/bmjopen-2015-008073 PMC4710818

[46] Heath L, Stevens R, Nicholson BD, Wherton J, Gao M, Callan C, et al. Strategies to improve the implementation of preventive care in primary care: a systematic review and meta-analysis. BMC Med. 2024;22(1):412. 39334345 10.1186/s12916-024-03588-5 PMC11437661

[47] Berendsen BA, Kremers SP, Savelberg HH, Schaper NC, Hendriks MR. The implementation and sustainability of a combined lifestyle intervention in primary care: mixed method process evaluation. BMC Fam Pract. 2015;16:37. 25880376 10.1186/s12875-015-0254-5 PMC4372167

[48] Carlfjord S, Lindberg M, Andersson A. Sustained use of a tool for lifestyle intervention implemented in primary health care: a 2-year follow-up. J Eval Clin Pract. 2013;19(2):327–34. 22332821 10.1111/j.1365-2753.2012.01827.x

[49] Proctor E, Luke D, Calhoun A, McMillen C, Brownson R, McCrary S, et al. Sustainability of evidence-based healthcare: research agenda, methodological advances, and infrastructure support. Implement Sci. 2015;10:88. 26062907 10.1186/s13012-015-0274-5 PMC4494699

[50] Powell BJ, Beidas RS, Lewis CC, Aarons GA, McMillen JC, Proctor EK, et al. Methods to Improve the Selection and Tailoring of Implementation Strategies. J Behav Health Serv Res. 2017;44(2):177–94. 26289563 10.1007/s11414-015-9475-6 PMC4761530

[51] Powell BJ, Fernandez ME, Williams NJ, Aarons GA, Beidas RS, Lewis CC, et al. Enhancing the Impact of Implementation Strategies in Healthcare: A Research Agenda. Front public health. 2019;7:3. 30723713 10.3389/fpubh.2019.00003 PMC6350272

[52] Nilsen P, Kirk JW, Gunnarsson KU, Thomas K. Tempering implementation optimism: distinguishing between efficacy and effectiveness in implementation research. Implement Sci Commun. 2025;6(1):90. 40849653 10.1186/s43058-025-00781-2 PMC12374263

[53] Flynn R, Cassidy C, Dobson L, Al-Rassi J, Langley J, Swindle J, et al. Knowledge translation strategies to support the sustainability of evidence-based interventions in healthcare: a scoping review. Implement Sci. 2023;18(1):69. 38049900 10.1186/s13012-023-01320-0 PMC10694920

[54] Wilson BE, Booth CM. Real-world data: bridging the gap between clinical trials and practice. EClinicalMedicine. 2024;78:102915. 39588211 10.1016/j.eclinm.2024.102915 PMC11585814

[55] Brown CH, Curran G, Palinkas LA, Aarons GA, Wells KB, Jones L, et al. An Overview of Research and Evaluation Designs for Dissemination and Implementation. Annu Rev Public Health. 2017;38:1–22. 28384085 10.1146/annurev-publhealth-031816-044215 PMC5384265

[56] Handley MA, Lyles CR, McCulloch C, Cattamanchi A. Selecting and Improving Quasi-Experimental Designs in Effectiveness and Implementation Research. Annu Rev Public Health. 2018;39:5–25. 29328873 10.1146/annurev-publhealth-040617-014128 PMC8011057

[57] McCambridge J, Witton J, Elbourne DR. Systematic review of the Hawthorne effect: new concepts are needed to study research participation effects. J Clin Epidemiol. 2014;67(3):267–77. 24275499 10.1016/j.jclinepi.2013.08.015 PMC3969247

