# Supplementary material for: Sustainability of implementation of health-promotion practice in primary healthcare: a non-randomized parallel group study
Source: BMC Health Serv Res. 2026 Jul 20;26:1006. doi: 10.1186/s12913-026-15103-y (PMC13390329; doi:10.1186/s12913-026-15103-y)
Supplement: Supplementary file 1 — Supplementary Material 1 [file 12913_2026_15103_MOESM1_ESM.docx]

# **Appendix 1.** Action plan: material for discussion with primary healthcare managers to support the sustainability of a health-promotive practice.

VISION AND “WHY”

- Set vision and goals for the health-promotive work together with the staff and update them on a regular basis
- Decide when and how the effect goals will be evaluated
- Is the “why” clear?
- Are the expectations clear?

STRUCTURE

*Coordinators*

- Have you appointed coordinators for each lifestyle habit?
- Do they have an assignment description?
- Do you have a plan if you need to replace a coordinator?
- Can the coordinators participate in the region’s network or in other continuing education?

*Routines, materials, and tools*

- Are the routines, materials, and tools updated, adequate, feasible ,and sufficient?
- Is anyone responsible for updating the routines, materials, and tools?
- Is the quick reference guide for the national guideline recommendations accessible?

*Competence*

- Do the staff know where to find the routines, material, and tools?
- Do the staff know who the coordinator(s) for a specific lifestyle habit is/are?
- Do you have one or more co-workers with competence to give a consultative conversation for alcohol overuse, physical inactivity, and prescription of physical activity?
- Do you have one or more co-workers who can give qualified consultative conversations on diet and tobacco use?
- Does everyone know how to use the lifestyle screening form?
- Are you using it for the right patients?
- Do the staff know how to work according to the clinical guidelines?
- Do the staff know how to correctly register health-promotive activities in the medical record?
- Is there a plan for how to continuously educate staff and maintain competence within health promotion?
- Is it known how to find and analyze the results in order to enable feedback to the staff?

PEOPLE (STAFF)

- Is the vision and “why” known and accepted?
- Is everyone on board?
- Do they know what the goals are?
- Are they involved and engaged?
- Do they know what part they play?
- Are they committed?
- Are further steps necessary to integrate health promotion in clinical practice for different professional groups?

STRATEGIES

- Involve the staff and create possibilities for participation
- Provide prerequisites to gain and keep competence
- Strengthen the feeling of relatedness (being “us”)
- Visualize health-promotive work
- Clarify that health promotion is prioritized and a natural part of the clinical work
- Communicate the vision – keep it alive
- Provide feedback on a regular basis
- Act on the results, revise your strategies
- Listen to feedback from the staff
- Appoint resources
- Introduce new staff to health-promotive work
