# Supplementary material for: Sustainability of implementation of health-promotion practice in primary healthcare: a non-randomized parallel group study
Source: BMC Health Serv Res. 2026 Jul 20;26:1006. doi: 10.1186/s12913-026-15103-y (PMC13390329; doi:10.1186/s12913-026-15103-y)
Supplement: Supplementary file 2 — Supplementary Material 2 [file 12913_2026_15103_MOESM2_ESM.docx]

**Appendix 2.** Recommendations directed to top managers participating in the Act in Time project.

Your support is important for sustainability and continued improvement of the primary healthcare centers’ proactive health-promoting practice. Below we have listed scientific recommendations on sustainability of practice change as well as what your staff, internal facilitators, and managers have highlighted as important from their perspectives to sustain the change.

This is especially emphasized in the scientific literature:

| Goal setting/indicators align well with the organization’s strategic aims regarding health promotion and disease prevention practice.   - For an example goals and indicators formulated in the healthcare organizational plan, regional strategy for development, standards and quality. |
| --- |
| To conduct constructive follow-ups, specifically for health-promotion practice, of how the managers work with the action plan and the primary healthcare centers’ results.   - Allocate responsibility and clarify *who* is responsible for the follow-ups, and *how* and *when* they shall be performed. |

In line with theories on sustainability, staff and managers also request that you:

| Continue to communicate with clarity and support the primary care in the health-promoting practice, in line with the primary care mission.   - It is essential that we work upstream. - It will take time before we see results. - We need support to prioritize health-promoting practice. |
| --- |
| Continue to actively listen to new ideas and requested support from the managers at the centers.   - The centers have ideas for how they can develop their health-promoting practice. |
| Continue to engage and involve the managers at the centers.   - Important to involve new managers in the health-promoting practice. |
| Show that you appreciate the managers for their efforts to strive for a health-promoting practice at the center, and ensure that they feel appreciated.   - Keep showcasing best practices from the centers. |
| Provide support to each center to develop easily accessible graphs displaying real-time outcomes for staff.   - Access to results allows for timely responses and interventions when required. |
