# Supplementary material for: Sustainability of implementation of health-promotion practice in primary healthcare: a non-randomized parallel group study
Source: BMC Health Serv Res. 2026 Jul 20;26:1006. doi: 10.1186/s12913-026-15103-y (PMC13390329; doi:10.1186/s12913-026-15103-y)
Supplement: Supplementary file 3 — Supplementary Material 3 [file 12913_2026_15103_MOESM3_ESM.docx]

**Appendix 3.** Descriptive statistics for the matched primary healthcare centers including the summer months (shaded).

| **Pair and study phase** | **Month** | **Healthcare visits** | | | **Sent out lifestyle screening forms** | | | **Health-promoting activities** | | **Mean uptake per 1000 visits** | | **Crude uptake difference**  **per 1000 visits** |
| --- | --- | --- | --- | --- | --- | --- | --- | --- | --- | --- | --- | --- |
|  |  | Intervent | | Control | Intervent | | Control | Intervent | Control | Intervent | Control |  |
| **Pair 1 baseline** | 1 | 2358 | | 2195 | 3 | | 0 | 75 | 138 | 31.81 | 62.87 | -31.06 |
|  | 2 | 2922 | | 3407 | 0 | | 0 | 55 | 88 | 18.82 | 25.83 | -7.01 |
|  | 3 | 1875 | | 1967 | 0 | | 0 | 36 | 100 | 19.20 | 50.84 | -31.64 |
|  | 4 | 1856 | | 1838 | 0 | | 0 | 54 | 89 | 29.09 | 48.42 | -19.33 |
|  | 5 | 2232 | | 2033 | 3 | | 0 | 108 | 157 | 48.39 | 77.23 | -28.84 |
|  | 6 | 2631 | | 2502 | 4 | | 0 | 149 | 175 | 56.63 | 69.94 | -13.31 |
|  | 7 | 2258 | | 2610 | 7 | | 0 | 81 | 153 | 35.87 | 58.62 | -22.75 |
|  | **Phase total** | **16132** | | **16552** | **17** | | **0** | **558** | **900** | **34.59** | **54.37** | **-19.78** |
| **Pair 1 active** | 1 | 2476 | | 2577 | 6 | | 0 | 72 | 141 | 29.08 | 54.71 | -25.64 |
|  | 2 | 2387 | | 2551 | 6 | | 0 | 73 | 80 | 30.58 | 31.36 | -0.78 |
|  | 3 | 1621 | | 1926 | 1 | | 0 | 11 | 27 | 6.79 | 14.02 | -7.23 |
|  | 4 | 2208 | | 2412 | 9 | | 0 | 36 | 52 | 16.30 | 21.56 | -5.25 |
|  | 5 | 2831 | | 3078 | 8 | | 0 | 113 | 139 | 39.92 | 45.16 | -5.24 |
|  | 6 | 2631 | | 2956 | 4 | | 0 | 91 | 181 | 34.59 | 61.23 | -26.64 |
|  | 7 | 2806 | | 3070 | 2 | | 0 | 136 | 157 | 48.47 | 51.14 | -2.67 |
|  | 8 | 2208 | | 2568 | 0 | | 0 | 88 | 92 | 39.86 | 35.83 | 4.03 |
|  | 9 | 2279 | | 2469 | 1 | | 0 | 114 | 45 | 50.02 | 18.23 | 31.80 |
|  | 10 | 2123 | | 2585 | 15 | | 0 | 99 | 99 | 46.63 | 38.30 | 8.33 |
|  | 11 | 2738 | | 3101 | 22 | | 0 | 166 | 127 | 60.63 | 40.95 | 19.67 |
|  | 12 | 2168 | | 2570 | 29 | | 0 | 140 | 120 | 64.58 | 46.69 | 17.88 |
|  | 13 | 2458 | | 2870 | 43 | | 0 | 178 | 157 | 72.42 | 54.70 | 17.71 |
|  | 14 | 2394 | | 2368 | 49 | | 0 | 160 | 90 | 66.83 | 38.01 | 28.83 |
|  | 15 | 1708 | | 1637 | 47 | | 0 | 53 | 18 | 31.03 | 11.00 | 20.03 |
|  | 16 | 2239 | | 2238 | 35 | | 0 | 134 | 49 | 59.85 | 21.89 | 37.95 |
|  | 17 | 2769 | | 2536 | 98 | | 0 | 190 | 104 | 68.62 | 41.01 | 27.61 |
|  | 18 | 2518 | | 2686 | 67 | | 0 | 177 | 124 | 70.29 | 46.17 | 24.13 |
|  | 19 | 2465 | | 2530 | 157 | | 0 | 150 | 145 | 60.85 | 57.31 | 3.54 |
|  | **Phase total** | **45027** | | **48728** | **599** | | **0** | **2181** | **1947** | **48.44** | **39.96** | **8.48** |
| **Pair 1 follow-up** | 1 | 2296 | | 2149 | 61 | | 0 | 209 | 87 | 91.03 | 40.48 | 50.54 |
|  | 2 | 2508 | | 2681 | 44 | | 0 | 261 | 176 | 104.07 | 65.65 | 38.42 |
|  | 3 | 2245 | | 2693 | 95 | | 0 | 218 | 140 | 97.10 | 51.99 | 45.12 |
|  | 4 | 2328 | | 2528 | 134 | | 0 | 252 | 169 | 108.25 | 66.85 | 41.40 |
|  | 5 | 1875 | | 1977 | 67 | | 0 | 196 | 113 | 104.53 | 57.16 | 47.38 |
|  | 6 | 2324 | | 2313 | 96 | | 0 | 207 | 113 | 89.07 | 48.85 | 40.22 |
|  | 7 | 2052 | | 1943 | 25 | | 0 | 158 | 90 | 77.00 | 46.32 | 30.68 |
|  | 8 | 1499 | | 1437 | 25 | | 0 | 61 | 29 | 40.69 | 20.18 | 20.51 |
|  | 9 | 2067 | | 2011 | 62 | | 0 | 175 | 111 | 84.66 | 55.20 | 29.47 |
|  | 10 | 2065 | | 2164 | 53 | | 0 | 155 | 128 | 75.06 | 59.15 | 15.91 |
|  | 11 | 2294 | | 2481 | 57 | | 0 | 164 | 148 | 71.49 | 59.65 | 11.84 |
|  | 12 | 2394 | | 2304 | 98 | | 0 | 242 | 103 | 101.09 | 44.70 | 56.38 |
|  | 13 | 1784 | | 1751 | 26 | | 0 | 126 | 46 | 70.63 | 26.27 | 44.36 |
|  | 14 | 2424 | | 2271 | 57 | | 0 | 129 | 83 | 53.22 | 36.55 | 16.67 |
|  | 15 | 2273 | | 2215 | 64 | | 0 | 149 | 71 | 65.55 | 32.05 | 33.50 |
|  | 16 | 2341 | | 2103 | 36 | | 6 | 127 | 77 | 54.25 | 36.61 | 17.64 |
|  | 17 | 2107 | | 2336 | 35 | | 10 | 109 | 108 | 51.73 | 46.23 | 5.50 |
|  | 18 | 2476 | | 2055 | 49 | | 12 | 143 | 73 | 57.75 | 35.52 | 22.23 |
|  | 19 | 1943 | | 1646 | 17 | | 0 | 144 | 34 | 74.11 | 20.66 | 53.46 |
|  | 20 | 1581 | | 1559 | 25 | | 0 | 62 | 14 | 39.22 | 8.98 | 30.24 |
|  | 21 | 2488 | | 2131 | 20 | | 0 | 104 | 46 | 41.80 | 21.59 | 20.21 |
|  | **Phase total** | **45364** | | **44748** | **1146** | | **28** | **3391** | **1959** | **74.75** | **43.78** | **30.97** |
| **Pair 2 baseline** | 1 | 1532 | 1273 | | 4 | 3 | | 119 | 98 | 77.68 | 76.98 | 0.69 |
|  | 2 | 1413 | 1174 | | 0 | 5 | | 184 | 54 | 130.22 | 46.00 | 84.22 |
|  | 3 | 1480 | 1267 | | 0 | 2 | | 170 | 83 | 114.86 | 65.51 | 49.36 |
|  | 4 | 1606 | 1237 | | 7 | 4 | | 177 | 76 | 110.21 | 61.44 | 48.77 |
|  | 5 | 1169 | 829 | | 5 | 0 | | 79 | 6 | 67.58 | 7.24 | 60.34 |
|  | 6 | 1209 | 993 | | 6 | 0 | | 60 | 54 | 49.63 | 54.38 | -4.75 |
|  | **Phase total** | **8409** | **6773** | | **22** | **14** | | **789** | **371** | **93.83** | **54.78** | **39.05** |
| **Pair 2 active** | 1 | 1532 | 1273 | | 4 | 3 | | 119 | 98 | 77.68 | 76.98 | 0.69 |
|  | 2 | 1594 | 1232 | | 29 | 3 | | 106 | 69 | 66.50 | 56.01 | 10.49 |
|  | 3 | 1689 | 1283 | | 33 | 1 | | 137 | 79 | 81.11 | 61.57 | 19.54 |
|  | 4 | 1471 | 953 | | 35 | 1 | | 126 | 56 | 85.66 | 58.76 | 26.89 |
|  | 5 | 1305 | 1059 | | 43 | 0 | | 132 | 50 | 101.15 | 47.21 | 53.94 |
|  | 6 | 1655 | 1252 | | 40 | 1 | | 162 | 9 | 97.89 | 7.19 | 90.70 |
|  | 7 | 1862 | 1497 | | 84 | 2 | | 258 | 61 | 138.56 | 40.75 | 97.81 |
|  | 8 | 1539 | 1081 | | 65 | 6 | | 177 | 78 | 115.01 | 72.16 | 42.85 |
|  | 9 | 1671 | 1186 | | 63 | 1 | | 284 | 30 | 169.96 | 25.30 | 144.66 |
|  | 10 | 1454 | 918 | | 60 | 0 | | 194 | 18 | 133.43 | 19.61 | 113.82 |
|  | 11 | 1015 | 667 | | 29 | 1 | | 92 | 6 | 90.64 | 9.00 | 81.64 |
|  | 12 | 1247 | 958 | | 29 | 0 | | 179 | 3 | 143.54 | 3.13 | 140.41 |
|  | 13 | 1375 | 1039 | | 35 | 7 | | 206 | 126 | 149.82 | 121.27 | 28.55 |
|  | 14 | 1473 | 1066 | | 41 | 8 | | 156 | 194 | 105.91 | 181.99 | -76.08 |
|  | 15 | 1569 | 1143 | | 32 | 6 | | 243 | 121 | 154.88 | 105.86 | 49.01 |
|  | **Phase total** | **22451** | **16607** | | **622** | **40** | | **2571** | **998** | **114.52** | **60.10** | **54.42** |
| **Pair 2 follow-up** | 1 | 1418 | 853 | | 35 | 5 | | 184 | 147 | 129.76 | 172.33 | -42.57 |
|  | 2 | 1541 | 1167 | | 63 | 8 | | 203 | 129 | 131.73 | 110.54 | 21.19 |
|  | 3 | 1479 | 1055 | | 50 | 1 | | 208 | 122 | 140.64 | 115.64 | 25.00 |
|  | 4 | 1345 | 1139 | | 64 | 3 | | 237 | 96 | 176.21 | 84.28 | 91.92 |
|  | 5 | 970 | 950 | | 45 | 4 | | 202 | 97 | 208.25 | 102.11 | 106.14 |
|  | 6 | 1146 | 1260 | | 24 | 0 | | 143 | 157 | 124.78 | 124.60 | 0.18 |
|  | 7 | 862 | 1020 | |  | 0 | | 93 | 120 | 107.89 | 117.65 | -9.76 |
|  | 8 | 698 | 736 | | 3 | 0 | | 28 | 79 | 40.11 | 107.34 | -67.22 |
|  | 9 | 1178 | 1014 | | 12 | 0 | | 155 | 56 | 131.58 | 55.23 | 76.35 |
|  | 10 | 1179 | 1168 | | 14 | 8 | | 154 | 118 | 130.62 | 101.03 | 29.59 |
|  | 11 | 1209 | 1308 | | 10 | 8 | | 149 | 147 | 123.24 | 112.39 | 10.86 |
|  | 12 | 1224 | 1291 | | 20 | 12 | | 142 | 127 | 116.01 | 98.37 | 17.64 |
|  | 34 | 911 | 1074 | | 16 | 4 | | 143 | 129 | 156.97 | 120.11 | 36.86 |
|  | 13 | 1145 | 1346 | | 15 | 9 | | 147 | 38 | 128.38 | 28.23 | 100.15 |
|  | 14 | 1272 | 1312 | | 19 | 0 | | 170 | 90 | 133.65 | 68.60 | 65.05 |
|  | 15 | 1126 | 1242 | | 11 | 4 | | 155 | 95 | 137.66 | 76.49 | 61.17 |
|  | 16 | 1087 | 1324 | | 14 | 3 | | 150 | 84 | 137.99 | 63.44 | 74.55 |
|  | 17 | 1081 | 1226 | | 14 | 2 | | 144 | 120 | 133.21 | 97.88 | 35.33 |
|  | 18 | 888 | 941 | | 4 | 2 | | 76 | 99 | 85.59 | 105.21 | -19.62 |
|  | 19 | 846 | 744 | | 5 | 2 | | 30 | 18 | 35.46 | 24.19 | 11.27 |
|  | 20 | 1185 | 1058 | | 15 | 4 | | 113 | 45 | 95.36 | 42.53 | 52.83 |
|  | **Phase total** | **23790** | **23228** | | **453** | **79** | | **3026** | **2113** | **127.20** | **90.97** | **36.23** |
| **Pair 3 baseline** | 1 | 2127 | 4232 | | 0 | 0 | | 24 | 136 | 11.28 | 32.14 | -20.85 |
|  | 3 | 2690 | 4454 | | 0 | 0 | | 36 | 89 | 13.38 | 19.98 | -6.60 |
|  | 3 | 2719 | 4722 | | 0 | 0 | | 39 | 96 | 14.34 | 20.33 | -5.99 |
|  | 4 | 2753 | 4557 | | 0 | 1 | | 38 | 32 | 13.80 | 7.02 | 6.78 |
|  | 5 | 2120 | 3170 | | 0 | 0 | | 54 | 4 | 25.47 | 1.26 | 24.21 |
|  | 6 | 2618 | 4283 | | 0 | 0 | | 39 | 87 | 14.90 | 20.31 | -5.42 |
|  | **Phase total** | **15027** | **25418** | | **0** | **1** | | **230** | **444** | **15.31** | **17.47** | **-2.16** |
| **Pair 3 active** | 1 | 2916 | 5132 | | 0 | 0 | | 15 | 144 | 5.14 | 28.06 | -22.92 |
|  | 2 | 2955 | 4890 | | 0 | 0 | | 35 | 158 | 11.84 | 32.31 | -20.47 |
|  | 3 | 3073 | 5516 | | 0 | 0 | | 69 | 127 | 22.45 | 23.02 | -0.57 |
|  | 4 | 2441 | 4657 | | 0 | 0 | | 57 | 73 | 23.35 | 15.68 | 7.68 |
|  | 5 | 2285 | 4021 | | 0 | 0 | | 29 | 92 | 12.69 | 22.88 | -10.19 |
|  | 6 | 2355 | 4382 | | 0 | 0 | | 28 | 85 | 11.89 | 19.40 | -7.51 |
|  | 7 | 3214 | 5337 | | 0 | 0 | | 62 | 121 | 19.29 | 22.67 | -3.38 |
|  | 8 | 2483 | 4419 | | 1 | 1 | | 62 | 81 | 24.97 | 18.33 | 6.64 |
|  | 9 | 2413 | 4734 | | 0 | 0 | | 48 | 70 | 19.89 | 14.79 | 5.11 |
|  | 10 | 2153 | 4161 | | 0 | 0 | | 44 | 72 | 20.44 | 17.30 | 3.13 |
|  | 11 | 1728 | 3038 | | 2 | 2 | | 25 | 30 | 14.47 | 9.87 | 4.59 |
|  | 12 | 2593 | 4207 | | 3 | 3 | | 20 | 78 | 7.71 | 18.54 | -10.83 |
|  | 13 | 2610 | 4616 | | 20 | 20 | | 54 | 146 | 20.69 | 31.63 | -10.94 |
|  | 14 | 2623 | 4767 | | 65 | 65 | | 63 | 206 | 24.02 | 43.21 | -19.20 |
|  | 15 | 2714 | 5117 | | 59 | 59 | | 151 | 266 | 55.64 | 51.98 | 3.65 |
|  | 16 | 2355 | 4137 | | 60 | 60 | | 134 | 154 | 56.90 | 37.23 | 19.68 |
|  | 17 | 2759 | 4754 | | 72 | 72 | | 146 | 150 | 52.92 | 31.55 | 21.37 |
|  | 18 | 2241 | 4294 | | 28 | 28 | | 98 | 154 | 43.73 | 35.86 | 7.87 |
|  | **Phase total** | **45911** | **82179** | | **310** | **310** | | **1140** | **2207** | **24.83** | **26.86** | **-2.03** |
| **Pair 3 follow-up** | 1 | 2696 | 4563 | | 50 | 50 | | 166 | 202 | 61.57 | 44.27 | 17.30 |
|  | 2 | 2045 | 3352 | | 40 | 40 | | 189 | 165 | 92.42 | 49.22 | 43.20 |
|  | 3 | 2469 | 3773 | | 47 | 47 | | 237 | 144 | 95.99 | 38.17 | 57.82 |
|  | 4 | 1938 | 3285 | | 28 | 28 | | 96 | 172 | 49.54 | 52.36 | -2.82 |
|  | 5 | 1565 | 2430 | | 8 | 8 | | 66 | 61 | 42.17 | 25.10 | 17.07 |
|  | 6 | 2016 | 3245 | | 6 | 2 | | 125 | 165 | 62.00 | 50.85 | 11.16 |
|  | 7 | 2208 | 3622 | | 29 | 13 | | 167 | 230 | 75.63 | 63.50 | 12.13 |
|  | 8 | 2418 | 3567 | | 15 | 2 | | 236 | 346 | 97.60 | 97.00 | 0.60 |
|  | 9 | 2129 | 3781 | | 13 | 0 | | 138 | 254 | 64.82 | 67.18 | -2.36 |
|  | 10 | 1761 | 2733 | | 8 | 0 | | 107 | 134 | 60.76 | 49.03 | 11.73 |
|  | 11 | 2394 | 3610 | | 22 | 0 | | 144 | 230 | 60.15 | 63.71 | -3.56 |
|  | 12 | 2415 | 3732 | | 26 | 22 | | 183 | 266 | 75.78 | 71.28 | 4.50 |
|  | 13 | 2267 | 3418 | | 13 | 32 | | 135 | 197 | 59.55 | 57.64 | 1.91 |
|  | 14 | 2161 | 3734 | | 16 | 37 | | 138 | 298 | 63.86 | 79.81 | -15.95 |
|  | 15 | 2008 | 3709 | | 15 | 13 | | 115 | 244 | 57.27 | 65.79 | -8.52 |
|  | 16 | 2290 | 2663 | | 12 | 3 | | 170 | 90 | 74.24 | 33.80 | 40.44 |
|  | 17 | 2286 | 2632 | | 13 | 0 | | 133 | 25 | 58.18 | 9.50 | 48.68 |
|  | 18 | 3435 | 3176 | | 17 | 0 | | 228 | 80 | 66.38 | 25.19 | 41.19 |
|  | **Phase total** | **40501** | **61025** | | **378** | **297** | | **2773** | **3303** | **68.47** | **54.13** | **14.34** |
| **Pair 4 baseline** | 1 | 1425 | 1431 | | 1 | 2 | | 104 | 42 | 72.98 | 29.35 | 43.63 |
|  | 2 | 1025 | 1141 | | 0 | 0 | | 32 | 3 | 31.22 | 2.63 | 28.59 |
|  | 3 | 1361 | 1431 | | 1 | 1 | | 43 | 20 | 31.59 | 13.98 | 17.62 |
|  | 4 | 1586 | 1696 | | 1 | 1 | | 161 | 49 | 101.51 | 28.89 | 72.62 |
|  | 5 | 1536 | 1680 | | 0 | 0 | | 104 | 35 | 67.71 | 20.83 | 46.88 |
|  | 6 | 1655 | 1660 | | 0 | 1 | | 99 | 23 | 59.82 | 13.86 | 45.96 |
|  | **Phase total** | **8588** | **9039** | | **3** | **5** | | **543** | **172** | **63.23** | **19.03** | **44.20** |
| **Pair 4 active** | 1 | 1311 | 1271 | | 0 | 2 | | 61 | 16 | 46.53 | 12.59 | 33.94 |
|  | 2 | 1327 | 1341 | | 15 | 2 | | 101 | 39 | 76.11 | 29.08 | 47.03 |
|  | 3 | 1443 | 1272 | | 7 | 2 | | 133 | 34 | 92.17 | 26.73 | 65.44 |
|  | 4 | 1907 | 1627 | | 9 | 1 | | 221 | 49 | 115.89 | 30.12 | 85.77 |
|  | 5 | 1509 | 1256 | | 8 | 4 | | 138 | 39 | 91.45 | 31.05 | 60.40 |
|  | 6 | 1655 | 1533 | | 8 | 4 | | 187 | 107 | 112.99 | 69.80 | 43.19 |
|  | 7 | 1420 | 1033 | | 6 | 1 | | 357 | 63 | 251.41 | 60.99 | 190.42 |
|  | 8 | 994 | 860 | | 5 | 0 | | 198 | 0 | 199.20 | 0.00 | 199.20 |
|  | 9 | 1319 | 1076 | | 13 | 0 | | 224 | 27 | 169.83 | 25.09 | 144.73 |
|  | 10 | 1519 | 1292 | | 2 | 0 | | 223 | 97 | 146.81 | 75.08 | 71.73 |
|  | 11 | 1649 | 1531 | | 15 | 0 | | 291 | 76 | 176.47 | 49.64 | 126.83 |
|  | 12 | 1623 | 1668 | | 59 | 0 | | 329 | 148 | 202.71 | 88.73 | 113.98 |
|  | 13 | 1357 | 1345 | | 35 | 0 | | 292 | 85 | 215.18 | 63.20 | 151.98 |
|  | 14 | 1375 | 1514 | | 56 | 0 | | 320 | 122 | 232.73 | 80.58 | 152.15 |
|  | 15 | 1434 | 1358 | | 50 | 0 | | 316 | 101 | 220.36 | 74.37 | 145.99 |
|  | **Phase total** | **21842** | **19977** | | **288** | **16** | | **3391** | **1003** | **155.25** | **50.21** | **105.04** |
| **Pair 4 follow-up** | 1 | 1647 | 1645 | | 52 | 0 | | 439 | 108 | 266.55 | 65.65 | 200.89 |
|  | 2 | 1306 | 1090 | | 35 | 0 | | 245 | 56 | 187.60 | 51.38 | 136.22 |
|  | 3 | 1509 | 1340 | | 23 | 1 | | 224 | 69 | 148.44 | 51.49 | 96.95 |
|  | 4 | 1266 | 1192 | | 23 | 2 | | 174 | 38 | 137.44 | 31.88 | 105.56 |
|  | 5 | 646 | 912 | | 6 | 0 | | 38 | 12 | 58.82 | 13.16 | 45.67 |
|  | 6 | 1334 | 1218 | | 45 | 1 | | 223 | 76 | 167.17 | 62.40 | 104.77 |
|  | 7 | 1418 | 1274 | | 43 | 2 | | 287 | 111 | 202.40 | 87.13 | 115.27 |
|  | 8 | 1471 | 1330 | | 72 | 1 | | 412 | 88 | 280.08 | 66.17 | 213.92 |
|  | 9 | 1548 | 1290 | | 48 | 2 | | 340 | 104 | 219.64 | 80.62 | 139.02 |
|  | 10 | 726 | 1062 | | 60 | 1 | | 204 | 53 | 280.99 | 49.91 | 231.09 |
|  | 11 | 1425 | 1405 | | 40 | 1 | | 282 | 46 | 197.89 | 32.74 | 165.15 |
|  | 12 | 1471 | 1253 | | 36 | 0 | | 307 | 37 | 208.70 | 29.53 | 179.17 |
|  | 13 | 1644 | 1196 | | 22 | 0 | | 350 | 32 | 212.90 | 26.76 | 186.14 |
|  | 14 | 1602 | 1292 | | 10 | 0 | | 211 | 35 | 131.71 | 27.09 | 104.62 |
|  | 15 | 1392 | 1318 | | 10 | 0 | | 214 | 18 | 153.74 | 13.66 | 140.08 |
|  | 16 | 1012 | 1071 | | 2 | 1 | | 107 | 18 | 105.73 | 16.81 | 88.92 |
|  | 17 | 862 | 1119 | | 3 | 0 | | 79 | 9 | 91.65 | 8.04 | 83.60 |
|  | 18 | 1157 | 1236 | | 5 | 0 | | 88 | 24 | 76.06 | 19.42 | 56.64 |
|  | **Phase total** | **23436** | **22243** | | **535** | **12** | | **4224** | **910** | **180.24** | **40.91** | **139.32** |
| **Pair 5 baseline** | 1 | 1593 | 1717 | | 2 | 19 | | 17 | 47 | 10.67 | 27.37 | -16.70 |
|  | 2 | 1891 | 2095 | | 3 | 44 | | 67 | 85 | 35.43 | 40.57 | -5.14 |
|  | 3 | 2014 | 2021 | | 4 | 43 | | 45 | 94 | 22.34 | 46.51 | -24.17 |
|  | 4 | 2134 | 2212 | | 5 | 45 | | 44 | 80 | 20.62 | 36.17 | -15.55 |
|  | 5 | 1909 | 1909 | | 2 | 28 | | 30 | 60 | 15.72 | 31.43 | -15.72 |
|  | 6 | 1943 | 1711 | | 0 | 25 | | 29 | 32 | 14.93 | 18.70 | -3.78 |
|  | **Phase total** | **11484** | **9453** | | **16** | **204** | | **232** | **398** | **20.20** | **42.10** | **-21.90** |
| **Pair 5 active** | 1 | 2048 | 1747 | | 0 | 11 | | 51 | 51 | 24.90 | 29.19 | -4.29 |
|  | 2 | 2511 | 2317 | | 1 | 10 | | 97 | 73 | 38.63 | 31.51 | 7.12 |
|  | 3 | 2075 | 1811 | | 12 | 13 | | 79 | 46 | 38.07 | 25.40 | 12.67 |
|  | 4 | 2479 | 2173 | | 21 | 17 | | 117 | 67 | 47.20 | 30.83 | 16.36 |
|  | 5 | 1897 | 2056 | | 7 | 7 | | 90 | 69 | 47.44 | 33.56 | 13.88 |
|  | 6 | 1515 | 1343 | | 1 | 3 | | 10 | 9 | 6.60 | 6.70 | -0.10 |
|  | 7 | 2059 | 1902 | | 17 | 9 | | 48 | 18 | 23.31 | 9.46 | 13.85 |
|  | 8 | 2341 | 1983 | | 17 | 22 | | 129 | 44 | 55.10 | 22.19 | 32.92 |
|  | 9 | 2291 | 1931 | | 108 | 9 | | 118 | 24 | 51.51 | 12.43 | 39.08 |
|  | 10 | 2341 | 2018 | | 153 | 26 | | 149 | 25 | 63.65 | 12.39 | 51.26 |
|  | 11 | 2072 | 1855 | | 109 | 8 | | 135 | 20 | 65.15 | 10.78 | 54.37 |
|  | 12 | 2378 | 1999 | | 98 | 5 | | 95 | 62 | 39.95 | 31.02 | 8.93 |
|  | 13 | 2098 | 1945 | | 127 | 1 | | 113 | 71 | 53.86 | 36.50 | 17.36 |
|  | 14 | 2019 | 2239 | | 147 | 14 | | 154 | 79 | 76.28 | 35.28 | 40.99 |
|  | 15 | 1628 | 1827 | | 105 | 9 | | 116 | 67 | 71.25 | 36.67 | 34.58 |
|  | **Phase total** | **31752** | **11665** | | **923** | **164** | | **1385** | **725** | **43.62** | **62.15** | **-18.53** |
| **Pair 5 follow-up** | 1 | 2109 | 2286 | | 67 | 7 | | 115 | 83 | 54.53 | 36.31 | 18.22 |
|  | 2 | 1816 | 2041 | | 36 | 2 | | 70 | 71 | 38.55 | 34.79 | 3.76 |
|  | 3 | 1291 | 1526 | | 105 | 1 | | 20 | 21 | 15.49 | 13.76 | 1.73 |
|  | 4 | 1827 | 2009 | | 100 | 7 | | 80 | 53 | 43.79 | 26.38 | 17.41 |
|  | 5 | 2028 | 2098 | | 203 | 10 | | 165 | 88 | 81.36 | 41.94 | 39.42 |
|  | 6 | 1957 | 1993 | | 166 | 13 | | 137 | 96 | 70.01 | 48.17 | 21.84 |
|  | 7 | 1974 | 1969 | | 135 | 26 | | 122 | 78 | 61.80 | 39.61 | 22.19 |
|  | 8 | 1570 | 1673 | | 97 | 8 | | 65 | 38 | 41.40 | 22.71 | 18.69 |
|  | 9 | 1995 | 1875 | | 114 | 11 | | 90 | 80 | 45.11 | 42.67 | 2.45 |
|  | 10 | 1906 | 2178 | | 147 | 8 | | 76 | 59 | 39.87 | 27.09 | 12.79 |
|  | 11 | 1899 | 1925 | | 94 | 8 | | 76 | 76 | 40.02 | 39.48 | 0.54 |
|  | 12 | 2035 | 1961 | | 106 | 7 | | 89 | 80 | 43.73 | 40.80 | 2.94 |
|  | 13 | 2112 | 2004 | | 63 | 3 | | 77 | 57 | 36.46 | 28.44 | 8.02 |
|  | 14 | 1591 | 1632 | | 12 | 1 | | 51 | 43 | 32.06 | 26.35 | 5.71 |
|  | 15 | 1324 | 1754 | | 57 | 1 | | 19 | 26 | 14.35 | 14.82 | -0.47 |
|  | 16 | 1976 | 2020 | | 29 | 4 | | 41 | 32 | 20.75 | 15.84 | 4.91 |
|  | **Phase total** | **29410** | **30944** | | **1531** | **117** | | **1293** | **981** | **43.96** | **31.70** | **12.26** |
