# Supplementary material for: Sustainability of implementation of health-promotion practice in primary healthcare: a non-randomized parallel group study
Source: BMC Health Serv Res. 2026 Jul 20;26:1006. doi: 10.1186/s12913-026-15103-y (PMC13390329; doi:10.1186/s12913-026-15103-y)
Supplement: Supplementary file 4 — Supplementary Material 4 [file 12913_2026_15103_MOESM4_ESM.docx]

**Appendix 4.** Physical activity on prescription for the participating healthcare centers during baseline, active implementation intervention and follow-up phases

|  | | **Baseline** | **Per 1000 visits at baseline** | **Active phase** | **Per 1000 visits in active phase** | **Follow-up phase** | **Per 100 visits during follow-up** |
| --- | --- | --- | --- | --- | --- | --- | --- |
| **Pair 1** | Intervention | 111 | 6.9 | 143 | 4.4 | 219 | 6.5 |
|  | Control | 44 | 2.7 | 166 | 4.7 | 178 | 5.2 |
| **Pair 2** | Intervention | 15 | 3.7 | 71 | 3.8 | 156 | 8.6 |
|  | Control | 28 | 7.5 | 35 | 2.5 | 9 | 0.5 |
| **Pair 3** | Intervention | 12 | 2.0 | 174 | 4.4 | 137 | 5.1 |
|  | Control | 20 | 1.5 | 249 | 3.5 | 11 | 0.3 |
| **Pair 4** | Intervention | 13 | 2.7 | 114 | 6.3 | 90 | 5.2 |
|  | Control | 11 | 2.2 | 26 | 1.5 | 7 | 0.5 |
| **Pair 5** | Intervention | 40 | 4.0 | 106 | 4.0 | 72 | 3.7 |
|  | Control | 43 | 4.5 | 80 | 8.0 | 63 | 3.2 |
