# Supplementary material for: Sustainability of implementation of health-promotion practice in primary healthcare: a non-randomized parallel group study
Source: BMC Health Serv Res. 2026 Jul 20;26:1006. doi: 10.1186/s12913-026-15103-y (PMC13390329; doi:10.1186/s12913-026-15103-y)
Supplement: Supplementary file 5 — Supplementary Material 5 [file 12913_2026_15103_MOESM5_ESM.docx]

**Appendix 5.** Predicted monthly uptake rate per 1000 visits by study group and lower and upper 95% confidence limits.

| **Intervention** | **Study month** | **Uptake** | **Asymptotic confidence limit** | |
| --- | --- | --- | --- | --- |
|  |  |  | **Lower** | **Upper** |
| No | -6 | 43.01 | 26.77 | 69.10 |
| Yes | -6 | 42.01 | 26.26 | 67.18 |
| No | -5 | 43.01 | 26.77 | 69.10 |
| Yes | -5 | 42.01 | 26.26 | 67.18 |
| No | -4 | 43.01 | 26.77 | 69.10 |
| Yes | -4 | 42.01 | 26.26 | 67.18 |
| No | -3 | 43.01 | 26.77 | 69.10 |
| Yes | -3 | 42.01 | 26.26 | 67.18 |
| No | -2 | 43.01 | 26.77 | 69.10 |
| Yes | -2 | 42.01 | 26.26 | 67.18 |
| No | -1 | 43.01 | 26.77 | 69.10 |
| Yes | -1 | 42.01 | 26.26 | 67.18 |
| No | 0 | 43.01 | 26.77 | 69.10 |
| Yes | 0 | 42.01 | 26.26 | 67.18 |
| No | 1 | 42.46 | 27.42 | 65.74 |
| Yes | 1 | 44.47 | 28.83 | 68.60 |
| No | 2 | 41.92 | 28.07 | 62.62 |
| Yes | 2 | 47.09 | 31.62 | 70.13 |
| No | 3 | 41.49 | 28.75 | 59.85 |
| Yes | 3 | 49.93 | 34.67 | 71.87 |
| No | 4 | 41.22 | 29.53 | 57.54 |
| Yes | 4 | 53.08 | 38.10 | 73.97 |
| No | 5 | 41.22 | 30.46 | 55.78 |
| Yes | 5 | 56.67 | 41.93 | 76.58 |
| No | 6 | 41.56 | 31.60 | 54.66 |
| Yes | 6 | 60.81 | 46.28 | 79.90 |
| No | 7 | 42.34 | 33.02 | 54.30 |
| Yes | 7 | 65.70 | 51.29 | 84.16 |
| No | 8 | 43.68 | 34.80 | 54.83 |
| Yes | 8 | 71.55 | 57.10 | 89.67 |
| No | 9 | 45.73 | 37.03 | 56.46 |
| Yes | 9 | 78.66 | 63.88 | 96.86 |
| No | 10 | 48.67 | 39.84 | 59.46 |
| Yes | 10 | 87.41 | 71.85 | 106.35 |
| No | 11 | 52.79 | 43.36 | 64.26 |
| Yes | 11 | 98.31 | 81.23 | 118.98 |
| No | 12 | 58.45 | 47.80 | 71.48 |
| Yes | 12 | 112.05 | 92.32 | 135.99 |
| No | 13 | 55.50 | 45.35 | 67.93 |
| Yes | 13 | 106.43 | 87.41 | 129.60 |
| No | 14 | 53.98 | 43.54 | 66.91 |
| Yes | 14 | 102.67 | 83.24 | 126.64 |
| No | 15 | 53.56 | 42.33 | 67.77 |
| Yes | 15 | 100.36 | 79.82 | 126.19 |
| No | 16 | 53.80 | 41.49 | 69.76 |
| Yes | 16 | 98.93 | 76.91 | 127.25 |
| No | 17 | 54.21 | 40.73 | 72.16 |
| Yes | 17 | 97.81 | 74.18 | 128.95 |
| No | 18 | 54.32 | 39.75 | 74.23 |
| Yes | 18 | 96.47 | 71.33 | 130.46 |
| No | 19 | 53.65 | 38.27 | 75.22 |
| Yes | 19 | 94.41 | 68.03 | 131.01 |
| No | 20 | 51.88 | 36.07 | 74.61 |
| Yes | 20 | 91.30 | 64.09 | 130.05 |
| No | 21 | 49.20 | 33.32 | 72.65 |
| Yes | 21 | 87.33 | 59.67 | 127.81 |
| No | 22 | 45.96 | 30.25 | 69.81 |
| Yes | 22 | 82.81 | 54.97 | 124.73 |
| No | 23 | 42.44 | 27.08 | 66.51 |
| Yes | 23 | 78.02 | 50.22 | 121.19 |
| No | 24 | 38.91 | 24.00 | 63.08 |
| Yes | 24 | 73.19 | 45.60 | 117.48 |
| No | 25 | 35.56 | 21.14 | 59.81 |
| Yes | 25 | 68.54 | 41.25 | 113.90 |
| No | 26 | 32.49 | 18.59 | 56.81 |
| Yes | 26 | 64.18 | 37.26 | 110.57 |
| No | 27 | 29.69 | 16.31 | 54.05 |
| Yes | 27 | 60.10 | 33.61 | 107.47 |
| No | 28 | 27.14 | 14.30 | 51.50 |
| Yes | 28 | 56.28 | 30.30 | 104.54 |
| No | 29 | 24.80 | 12.52 | 49.12 |
| Yes | 29 | 52.70 | 27.29 | 101.78 |
| No | 30 | 22.66 | 10.95 | 46.89 |
| Yes | 30 | 49.35 | 24.56 | 99.16 |
