# Supplementary material for: Sustainability of implementation of health-promotion practice in primary healthcare: a non-randomized parallel group study
Source: BMC Health Serv Res. 2026 Jul 20;26:1006. doi: 10.1186/s12913-026-15103-y (PMC13390329; doi:10.1186/s12913-026-15103-y)
Supplement: Supplementary file 6 — Supplementary Material 6 [file 12913_2026_15103_MOESM6_ESM.docx]

**Appendix 6.** Rate of rate ratios and lower and upper confidence limits per study month.

| **Months since study start** | **Ratio** | **Asymptotic confidence limits** | |
| --- | --- | --- | --- |
|  |  | **Lower** | **Upper** |
| 0 | 1 | 1 | 1 |
| 0 | 1 | 1 | 1 |
| 1 | 1.0724702457423834 | 1.0433652363590105 | 1.1023871487385457 |
| 2 | 1.1500496145739696 | 1.0886001300640298 | 1.2149678099927672 |
| 3 | 1.2323223830438945 | 1.135723858732555 | 1.3371370549930428 |
| 4 | 1.3185146739569193 | 1.184710957042889 | 1.4674304606577422 |
| 5 | 1.4075858885322377 | 1.235458953454761 | 1.6036939374268238 |
| 6 | 1.498202724212661 | 1.2877241109975768 | 1.7430840842914543 |
| 7 | 1.5887239308583276 | 1.3410220002910105 | 1.882179209538846 |
| 8 | 1.6772001955708737 | 1.394472303872888 | 2.017250889967761 |
| 9 | 1.761393619666504 | 1.4465603859301632 | 2.1447479922567516 |
| 10 | 1.8388208358849074 | 1.4947907134343774 | 2.262030420777638 |
| 11 | 1.9068227798909967 | 1.5352487086227453 | 2.36832839753568 |
| 12 | 1.9626623912194083 | 1.5622296034259335 | 2.4657346483894833 |
| 13 | 1.9634226980548144 | 1.5780246935841311 | 2.4429457326684854 |
| 14 | 1.947510918325153 | 1.5622900879069943 | 2.427717365906678 |
| 15 | 1.9184375417755954 | 1.5267226592354273 | 2.410655648182435 |
| 16 | 1.882771758579688 | 1.4839423374698337 | 2.388791939819772 |
| 17 | 1.8472877250937043 | 1.444238684236989 | 2.3628171551745467 |
| 18 | 1.8182888804278088 | 1.4154393183842213 | 2.3357938484155856 |
| 19 | 1.8017200084698002 | 1.4031431710096889 | 2.313516579056209 |
| 20 | 1.801916498768756 | 1.408948610162025 | 2.3044865122239395 |
| 21 | 1.8172757321387614 | 1.4259786242665515 | 2.315947118996331 |
| 22 | 1.8449470698359338 | 1.4442039239865956 | 2.356889933590702 |
| 23 | 1.8821837849989336 | 1.4544744740268833 | 2.4356672212367974 |
| 24 | 1.9261641282592925 | 1.4519274055127889 | 2.555298725615384 |
| 25 | 1.9736509307659245 | 1.4369171974164916 | 2.7108715822434024 |
| 26 | 2.022423172952633 | 1.4126815585145145 | 2.895341463080174 |
| 27 | 2.0724006594765414 | 1.382473756787947 | 3.106637266936255 |
| 28 | 2.1236131739573354 | 1.3485803665224623 | 3.3440594454405717 |
| 29 | 2.1760912360181566 | 1.312534039104506 | 3.6078097225621666 |
| 30 | 2.2298661194734906 | 1.2753538583620716 | 3.8987633731407363 |
