# Supplementary figures and images for: Sustainability of implementation of health-promotion practice in primary healthcare: a non-randomized parallel group study
Source: BMC Health Serv Res. 2026 Jul 20;26:1006. doi: 10.1186/s12913-026-15103-y (PMC13390329; doi:10.1186/s12913-026-15103-y)

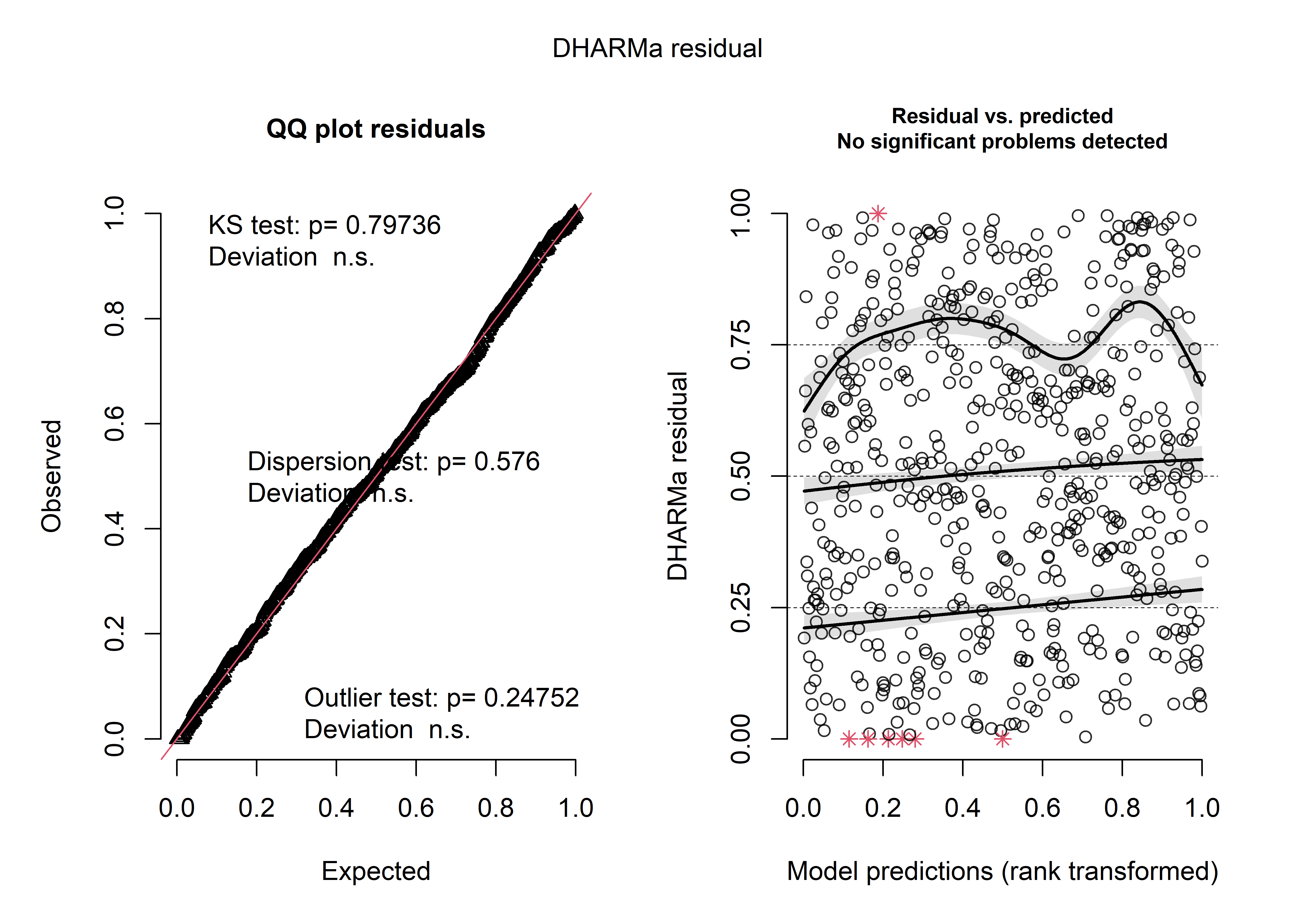

Supplement: Supplementary file 7 — Supplementary Material 7 [file 12913_2026_15103_MOESM7_ESM.tiff]
